# iPSCs‐derived iMSCs prevent osteoporotic bone loss and affect bone metabolites in ovariectomized mice

**DOI:** 10.1111/jcmm.70200

**Published:** 2024-11-24

**Authors:** Wei‐Zhou Wang, Yang‐Hao Wang, Sha‐Sha Bao, Fei He, Guoyu Li, Guang Yang, Jing Chen, Xin‐Yu Yang, Ya Xiao, Ya‐Shuang Tong, Xue‐Ting Zhao, Jun Hu, Ding‐You You

**Affiliations:** ^1^ Yunnan Provincial Key Laboratory of Public Health and Biosafety and School of Public Health The First Affiliated Hospital of Kunming Medical University Kunming Yunnan China; ^2^ Department of Orthopedics The First Affiliated Hospital of Kunming Medical University Kunming Yunnan China; ^3^ Department of Pathology The First Affiliated Hospital of Kunming Medical University Kunming Yunnan China; ^4^ Department of Radiology Yan'an Hospital Affiliated to Kunming Medical University Kunming Yunnan China; ^5^ Department of Orthopedics Kunming Medical University Affiliated Qujing Hospital Qujing Yunnan China; ^6^ Department of Colorectal Surgery, Yunnan Cancer Hospital The Third Affiliated Hospital of Kunming Medical University Kunming Yunnan China; ^7^ Kunming Medical University Kunming Yunnan China; ^8^ Trauma Medicine Centre The First Affiliated Hospital of Kunming Medical University Kunming Yunnan China; ^9^ Department of Pathology and Pathophysiology, Faculty of Basic Medical Science Kunming Medical University Kunming Yunnan China; ^10^ Department of Orthopedics Kunming First People's Hospital Kunming Yunnan China; ^11^ Yunnan Provincial Key Laboratory of Public Health and Biosafety and School of Public Health Kunming Medical University Kunming Yunnan China

**Keywords:** bone formation, cell transplantation, iMSCs, metabolites, osteoporosis

## Abstract

Osteoporosis is a metabolic bone disease that seriously jeopardizes the health of middle‐aged and elderly people. Mesenchymal stem cell‐based transplantation for osteoporosis is a promising new therapeutic strategy. Induced mesenchymal stem cells (iMSCs) are a new option for stem cell transplantation therapy. Acquired mouse skin fibroblasts were transduced and reprogrammed into induced pluripotent cells and further induced to differentiate into iMSCs. The iMSCs were tested for pluripotency markers, trilineage differentiation ability, cell surface molecular marker tests, and gene expression patterns. The iMSCs were injected into the tail vein of mice by tail vein injection, and the distribution of cells in various organs was observed. The effect of iMSCs on the bone mass of mice was detected after injection into the mouse osteoporosis model. The effects of iMSCs infusion on metabolites in femoral tissue and peripheral blood plasma were detected based on LC–MS untargeted metabolomics. iMSCs have similar morphology, immunophenotype, in vitro differentiation potential, and gene expression patterns as mesenchymal stem cells. The iMSCs were heavily distributed in the lungs after infusion and gradually decreased over time. The iMSCs in the femoral bone marrow cavity gradually increased with time. iMSCs infusion significantly avoided bone loss due to oophorectomy. The results of untargeted metabolomics suggest that amino acid and lipid metabolic pathways are key factors involved in iMSCs bone protection and prevention of osteoporosis formation. iMSCs obtained by reprogramming‐induced differentiation had cellular properties similar to those of bone marrow mesenchymal stem cells. The iMSCs could promote the remodelling of bone structure in ovariectomy‐induced osteoporotic mice and affect the changes of several key metabolites in bone and peripheral blood. Some of these metabolites can serve as potential biomarkers and therapeutic targets for iMSCs intervention in osteoporosis. Investigating the effects of iMSCs on osteoporosis and the influence of metabolic pathways will provide new ideas and methods for the clinical treatment of osteoporosis.

## 1 INTRODUCTION

Osteoporosis is a disease characterized by low bone mineral content and degeneration of bone tissue microstructure. It is not only the most common chronic metabolic bone disease but also a serious health hazard for middle‐aged and elderly people.^1^, ^2^ The prevalence of osteoporosis in men and women over the age of 50 is 6.3% and 21.2%, respectively, and based on the current global population, it is estimated that approximately 500 million people may be affected by the disease.^3^ In the United States alone, approximately 10 million men and women have been diagnosed with osteoporosis, and this number is growing.^4^ The basic intervention and drug treatment of osteoporosis are very important, and the ultimate goal is to prevent the aggravation of osteoporosis and the occurrence of osteoporotic fractures. At present, the pathogenesis of osteoporosis is still unclear. Clinical treatment of osteoporosis mainly involves anti‐bone resorption drugs and bone synthesis metabolic drugs. Although more emerging treatments and drugs for osteoporosis have been developed, the development of stem cell therapy and regenerative medicine provides a new approach and hope for the treatment of osteoporosis.

It has been proven that the failure and abnormal differentiation of stem cells in vivo is closely related to the occurrence of osteoporosis.^5^, ^6^, ^7^ Stem cell‐based therapy is becoming increasingly important in the treatment of chronic and long‐term diseases, including osteoporosis.^8^, ^9^, ^10^ Bone marrow mesenchymal stem cells (BMSCs) have been widely studied and applied in the treatment of osteoporosis because of their high differentiation ability and tissue regeneration and repair.^11^, ^12^, ^13^, ^14^ However, the efficacy of BMSCs is closely related to donor age, cell status and expansion generations, while BMSCs infusion is mostly from allogeneic sources, which have a potential risk of immune rejection, which limits the clinical application of stem cells.^15^

Induced pluripotent stem cells (iPSCs) can be constructed from arbitrary cells in the whole body and have good differentiation ability. The biological functions of Induced mesenchymal stem cells (iMSCs) derived from iPSCs are similar to those of mesenchymal stem cells. In theory, they can not only solve the problem of autologous donors but also provide a large number of expanded cells, which can fully meet the needs of clinical research.^16^ IPSCs‐derived iMSCs transplantation therapy has been preliminarily verified in disease models, including desiccation syndrome,^17^ cutaneous wound healing,^18^ steroid‐associated femoral head necrosis,^19^ and bone defects.^20^ However, there are few reports on the potential osteogenesis and chemotactic homing of iMSCs in the treatment of osteoporosis.

The purpose of our study was to clarify the similarities and differences between iPSCs‐derived iMSCs and BMSCs in cell biology, to further explore the distribution and chemotaxis of iMSCs via veins in vivo, to verify the role and osteogenic mechanism of iMSCs transplantation in osteoporosis and to analyse the changes in metabolites. The results of this study lay the foundation for revealing the safety and efficacy of iMSCs in the future treatment of osteoporosis and provide new insights into the clinical translation and application of iPSCs‐derived iMSCs.

## 2 METHODS

### 2.1 Acquisition of mouse skin fibroblasts

The bilateral ear tissues of C57BL/6 female mice (8 weeks old, weight 20 ± 3 g) were obtained and washed repeatedly with phosphate buffered saline (PBS) after soaking in 75% ethanol. The skin tissue was cut into pieces less than 1 mm^3^, digested with 0.25% trypsin, transferred to a culture bottle containing high sugar DMEM (Gibco, USA) and 10% fetal bovine serum (FBS) (Gibco, USA), and cultured at 37°C and 5% CO_2_. On the 3rd‐5th day, the cells around the tissue fragments grew radially and were digested by TrypLE Express (Gibco, USA) and subcultured.

### 2.2 Induction from fibroblasts to iPSCs

The mouse skin fibroblasts (Fibs) were reprogrammed according to the CytoTuneTM‐iPS 2.0 SV (Invitrogen, USA) program. After calculating the volume according to a multiplicity of infection (MOI) = 5:5:3 (KOS:c‐Myc:Klf4), Fibs containing 3 × 10^5^ cells were added for reprogramming. The 2nd–6th day was cultured in fresh DMEM containing 10% FBS, 1 × MEM Non‐Essential Amino Acids Solution (Gibco, USA), and 0.1% 2‐mercaptoethanol (Gibco, USA). On the 7th day, the reprogrammed cells were digested and plated on the inactivated feeder layer treated with mitomycin C for culture. DMEM/F‐12 GlutaMAX Supplement (Gibco, USA) containing 10% KnockOut Serum Replacement Multi‐Species (Gibco, USA), 1 × MEM Non‐Essential Amino Acids Solution, 0.1% 2‐mercaptoethanol and 10 μg/mL LIF Recombinant Mouse Protein (Gibco, USA) was used every 2–3 days. On the 16th–21st day, the colonies were identified by alkaline phosphatase (ALP) active staining (Invitrogen, USA). The fluorescence‐labelled colonies were observed by white light and FITC channel under a fluorescence microscope (Nikon, Japan). The colony was picked out with a needle and transferred to a new culture medium for amplification. When the iPSCs colony reaches approximately 60%–80% confluence, it will be subcultured.

### 2.3 Immunofluorescence staining

Fibs or iPSCs were cultured in a 4‐well chamber slide (Thermo Scientific, USA) and fixed with 4% paraformaldehyde, and 0.1% Triton X‐100 (Solarbio, China) was added for osmosis. Bovine serum albumin (BSA) (Solarbio, China) was added at room temperature for 30 min. Fibs were incubated with vimentin (2 μg/mL, abcampene, UK) and cytokeratin 19 (1:500, Abcam, UK), and iPSCs were incubated with Oct4 (1:250, Abcam, UK), Nanog (1:500, CST, UK), SOX2 (1:200, Abcam, USA) and SSEA‐4 (1:200, Abcam, UK) primary antibodies overnight at 4°C. The primary antibody was removed, and the sections were washed and incubated at room temperature for 1 h with an anti‐mouse fluorescence secondary antibody (1:1000, Proteintech, China) and an anti‐rabbit fluorescence secondary antibody (1:1000, Proteintech, China). Nuclei were stained with DAPI, and the sections were sealed with an anti‐quenching agent (Meilunbio, China). The staining results were observed, and the fluorescence results were photographed by laser confocal microscopy (Nikon, Japan).

### 2.4 Transdifferentiation from iPSCs to iMSCs

The iPSCs were planted in a nonadhesive coated plate without a feeder layer at a rate of 1 × 10^5^/mL. On the 2nd day, embryoid (EB) was induced by low‐sugar DMEM culture medium containing KnockOut Serum Replacement Multi‐Species, 1 × MEM Non‐Essential Amino Acids Solution, and 0.1% 2‐mercaptoethanol. To compare the effects of different induction methods on the production efficiency of iMSCs, they were divided into the control group (Ctrl‐iMSCs), transforming growth factor‐β (TGF‐β) group (TGF‐β‐iMSCs), basic fibroblast growth factor (bFGF) group (bFGF‐iMSCs), and TGF‐β1 and bFGF group (TGF‐β1/bFGF‐iMSCs). From the 8th day, EB was transferred to a 1% gelatin‐coated cell culture plate to continue culture. Ctrl‐iMSCs were induced in low‐sugar DMEM containing 10% FBS, TGF‐β‐iMSCs were induced in low‐sugar DMEM containing 10 ng/mL TGF‐β recombinant protein and 10% FBS, and bFGF‐iMSCs were induced in low‐sugar DMEM containing 5 ng/mL bFGF recombinant protein and 10% FBS. TGF‐β1/bFGF‐iMSCs were induced with 10 ng/mL recombinant TGF‐β1 protein, 5 ng/mL recombinant bFGF protein and 10% FBS in low‐glucose DMEM. The number of EB adherent cells and free cells per unit area were observed and recorded. On the 16th day, low‐sugar DMEM containing 10% FBS was added to all groups. When the free cells fused, TrypLE Express was used for digestion and subcultured.

### 2.5 Real‐time fluorescence quantitative PCR

The iPSCs and iMSCs grown in the logarithmic phase were cleaved according to the instructions of the TRIzol kit (Invitrogen, USA), and cell RNA was obtained by the centrifugal column method according to the procedure of the RNA extraction kit (Tiangen, China). The concentration and purity of RNA were determined by a NanoDrop One micro‐UV–Vis spectrophotometer. Template RNA (20 μL) was synthesized from 2 μg of total cDNA by a HiScript 1st Strand cDNA Synthesis Kit (Vazyme, China). Using the FastStart Universal SYBR Green Master (Roche, Switzerland) kit, each gene was amplified with 3 μL template cDNA and 2.4 μmol/L primers. Denatured at 95°C for 40 cycles and annealed at 60°C for 1 min. The specific primer sequences can be found in Table S1. The relative expression level of the target gene was calculated by 2^−∆∆ct^.

### 2.6 Flow cytometry analysis

The stably passaged iMSCs were digested and dispersed into single cells, and an adequate amount of resuspended cells was added to CD16/CD32 (1 μg per 10^6^ cells in 100 μL volume, BD, USA) for blocking. After centrifugation, under light‐avoiding conditions, the cells were incubated with anti‐CD29 APC (0.25 μg per 10^6^ cells in 100 μL volume, Biolegend, USA), anti‐Sca‐1‐PE‐Cy7 (0.25 μg per 10^6^ cells in 100 μL volume, Biolegend, USA), anti‐CD45 PE/Cyanine5 (0.25 μg per 10^6^ cells in 100 μL volume, Biolegend, USA), and anti‐CD11b‐PE (0.55 μg per 10^6^ cells in 100 μL volume, Biolegend, USA) for 30 min. After washing with PBS and resuspension, a flow cytometer (BD FACSAria, USA) was used for detection within 1 h. Flow Jo software (version V10.0) was used to analyse the data.

### 2.7 Cell proliferation and osteogenic and adipogenic differentiation

5‐Bromo‐2‐deoxyuridine (BrdU, Thermo Fisher, USA) was added to BMSCs and iMSCs and incubated for 48 h. The cells were fixed with 4% paraformaldehyde and permeabilized with 0.1% Triton X‐100. The cells were incubated with BrdU primary antibody (1:200, Cell Signalling, USA) overnight at 4°C. Remove the primary antibody and wash before adding a fluorescent secondary antibody against rabbit (1:1000, Proteintech, China) and incubating at room temperature for 1 h. DAPI was added for nuclear staining, and the slides were sealed with an anti‐quenching agent. A confocal laser microscope was used to observe and capture the staining results.

According to the procedure of the CCK8 cell proliferation detection kit (Tongren, Japan), the proliferation of BMSCs and iMSCs was detected on 0, 1, 3, 5 and 7 days. According to the induction procedures of the osteogenic differentiation kit (OriCell, China) and adipogenic differentiation kit (OriCell, China), Alizarin Red staining and Oil Red O staining were performed on Fibs, BMSCs and iMSCs cultured for 14 days to verify the osteogenic and adipogenic differentiation capabilities of the cells.

### 2.8 Mouse teratoma experiment

Resuspend passaged iPSCs and iMSCs and mix with Matrigel Matrix substrate gel (Corning, USA). An 8‐week‐old BALB/c‐nu male nude mouse was injected with 2 × 10^6^ cells into the subcutaneous axilla of the mouse. The nude mice were kept under standard conditions (12 h light and dark cycle, temperature 18–22°C, humidity 55% ± 5%). The nude mice were fed standard mouse feed and allowed ad libitum access to water, the growth of axillary tumours was regularly observed. When subcutaneous cell implantation was approximately 6 weeks old, the mice were euthanized by cervical dislocation, and photos were taken. Surgical instruments were used to separate the subcutaneous implantation site, and the cells were fixed with 4% paraformaldehyde for 72 h. After fixation, the tissue was dehydrated in a gradient, cleared, embedded in paraffin and then sliced on a paraffin slicer to a thickness of 10 μm. After dewaxing and rehydration, an HE staining kit was used for staining, followed by xylene for transparency and neutral balsam for sealing. The staining results were observed and photographed under an optical microscope.

### 2.9 Cell transcriptome sequencing and analysis

To compare the expression patterns of Fibs, iPSCs and iMSCs throughout the induction process, transcriptome sequencing was performed on each type of cell in three independent cultures using TRIzol reagent to extract total RNA. High‐quality RNA samples with RIN values >7.0 were used to construct a sequencing library. All library construction and sequencing work was assisted by Lianchuan Bio. Samples were sequenced on an Illumina NovaSeq™ 6000 with 2 × 150 bp paired‐end sequencing. After the final transcriptome was generated, StringTie and Ballgown (http://www.bioconductor.org/packages/release/bioc/html/ballgown.html) were used to estimate the expression levels of all transcripts and perform FPKM value calculations for mRNA expression abundance. DESeq2 software was used to perform differential gene expression analysis between the two different groups. Genes with a false discovery rate (FDR) parameter below 0.05 and an absolute fold change (logFC) >2 were considered differentially expressed genes (DEGs). Then, GO function and KEGG pathway enrichment analyses were performed on the differentially expressed genes. The princomp function in R (http://www.r‐project.org/) was used to perform principal component analysis (PCA).

### 2.10 Cell labeling and implantation

In combination with previous research reports,^21^, ^22^, ^23^ stably passaged BMSCs and iMSCs were resuspended at a concentration of 1 × 10^6^/mL, DiD staining reagent (Thermo Fisher, USA) was added at a volume of 5 μL into a cell suspension of 1 mL, incubated for 20 min and then washed repeatedly with PBS. We used an 8‐week‐old C57BL/6 mouse that had undergone ovariectomy (OVX) for 7 days and separately injected DiD‐BMSCs and DiD‐iMSCs at a cell count of 2 × 10^6^/200 μL into the mouse via tail vein injection. At 4 h, 1, 7, and 14 days after cell infusion, three mice were euthanized each time by cervical dislocation, and the mouse's heart, lungs, kidneys, spleen, liver, brain and bilateral femurs were quickly removed and fixed with 4% paraformaldehyde. After femur fixation, decalcification treatment was performed with 0.5 M EDTA. After fixation, the tissue was dehydrated with 20% sucrose for 24 h, embedded in OCT embedding agent (SAKURA, Japan) for freezing and prepared into slices of 20 μm thickness on a freezing microtome (Thermo Fisher, Japan). Nuclear staining with DAPI was performed, and the slides were sealed with a fluorescence quenching agent. A confocal laser microscope was used to observe and capture the staining results. The excitation wavelength parameters were 340 and 594 nm, and the results were finally analysed and quantified using ImageJ 3.0 software.

### 2.11 Mouse osteoporosis model construction and cell Implantation

Eight‐week‐old C57BL/6 female mice (weight 20 ± 3 g) were randomly divided into four groups: the Sham group, OVX group, BMSCs group and iMSCs group; each group contained 12 mice. To establish an osteoporosis model after OVX, the mice were anaesthetised intraperitoneally with 3% pentobarbital sodium (Kermel, China). The mice were shaved on their backsides; through bilateral dorsal entrances, the bilateral ovaries and part of the fallopian tubes of the OVX group, BMSCs group and iMSCs group were removed; then, they were sutured and disinfected. For the Sham group, only the ovaries were exposed, and a small amount of fat near the ovaries was removed; the rest of the surgical procedure was the same. During and after surgery, the mice were placed on a small animal heating blanket until they woke up. Feed them standard mouse feed and allow ad libitum access to water. After 7 and 21 days of model construction, tail vein infusion was performed on the BMSCs group and iMSCs group; each time, BMSCs and iMSCs were infused at a cell count of 2 × 10^6^ per mouse. For each mouse in the Sham group and OVX group, an equivalent volume of PBS was injected via the tail vein. On day 35 postsurgery, the mice were euthanized by cervical dislocation, and mouse plasma and bilateral femurs were obtained.

### 2.12 Micro‐CT evaluation and mechanical evaluation

The NEMO small animal high‐resolution imaging CT system (Pingsheng Medical, China) was used to scan and analyse mouse femurs. The x‐ray source tube pressure is set to 90 kV, the current is 60 μA, the frame rate is 20, DSD is set to 395 mm, Cruiser software is used for scanning and reconstruction, the CT reconstruction algorithm is FDK, the CT field of view is 15 mm, the pixel size is 0.0146 mm, and the slice thickness is 0.025 mm. After the scanning data were reconstructed, the region of interest was selected to analyse the main parameters of trabecular bone and cortical bone. Avatar3 software was used to analyse mouse femurs, with the proximal growth plate of the femur as a reference point. The metaphyseal region was selected, and the bone volume/density volume (BV/TV), number (*N*), thickness (Th) and bone mineral density (BMD) of trabecular bone (Tb) were mainly measured. The middle of the femur was selected to measure the BMD, Th, bone volume (BV) and bone area to total area ratio (Ar/Tt. Ar) of cortical bone (Ct).

Mouse femurs soaked in 4% paraformaldehyde were placed on a universal mechanical testing machine (HY‐0230, Shanghai Hengyi Precision Instrument Co., Ltd., China) to measure the biomechanical properties of femurs. The parameter settings are as follows: the diameter of the pressure head is 5 mm, the loading speed is 2 mm/min, and the span is 10 mm. The acquisition computer records the elastic load, maximum displacement, breaking load and stiffness. The mouse femur immersed in 4% paraformaldehyde was placed on a universal mechanical testing machine (HY‐0230, Shanghai Hengyi Precision Instrument Co., Ltd., China) to measure the biomechanical properties of the femur. The parameters were set as follows: the diameter of the indenter was 5 mm, the loading speed was 2 mm/min, and the span was 10 mm. The elastic load, maximum displacement, breaking load and stiffness were recorded by the acquisition computer.

### 2.13 Histological staining analysis of mouse femur pathology

After the completion of decalcification, the femur was embedded in paraffin wax after gradient dehydration, transparency and translucent wax embedding. Sections were made in the sagittal position at a thickness of 10 μm, rehydrated by dewaxing, stained with an HE kit (Solarbio, China) and Masson staining kit (Solarbio, China), and sealed with xylene clear and neutral gum. Decalcified femurs were immersed in 20% sucrose for 24 h. The staining results were observed and photographed using a light microscope (Olympus, Japan). The histological results were analysed using ImageJ software (version 2.0).

### 2.14 Nontargeted metabolomics testing and analysis

Acquired mouse femur tissues were accurately weighed to 100 mg (±2%), added to 1 mL of tissue extract (75% 9:1 methanol:chloroform, 25% H_2_O) (−20°C), and placed in a high‐throughput tissue grinder (Scientz, Ningbo, China) for grinding (55 Hz, 60 s) and centrifugation (12,000 rpm, 4°C, 10 min). The supernatant obtained after centrifugation was concentrated to dryness in a vacuum concentrator (Eppendorf, Germany). The samples were resolubilized by adding 200 μL of 50% acetonitrile solution configured as 2‐chlorophenylalanine solution (4 ppm) and filtered through a 0.22 μm membrane. The plasma samples were thawed and vortexed to mix well, an appropriate amount of sample was pipetted and vortexed with 400 μL of methanol solution for 1 min, and all the supernatant was extracted and filtered through a 0.22 μm membrane. The bone tissue and plasma filtrates were added to the assay vials and subjected to LC–MS. The LC–MS procedure and assay parameters, quality control and analysis of the raw metabolomics data were performed using a previously published paper.^24^

### 2.15 Data processing and statistical analysis

GraphPad Prism software V 9.0 was used for data analysis and visualization. The Shapiro–Wilk test and PP and QQ plots were used to determine the normality of the data, and data that conformed to a normal distribution were expressed as the mean ± SD. For data that conformed to a normal distribution and homogeneity, analysis of variance (ANOVA) was used to compare the overall mean differences between multiple groups of data, and then the least significant difference (LSD) test was performed on significantly different data to test the differences between the two groups. For data that did not conform to a normal distribution, the Kruskal–Wallis test was used to compare the statistical significance of the groups. *p* < 0.05 indicated that the difference was statistically significant.

## 3 RESULTS

### 3.1 Acquired skin Fibs reprogrammed into iPSCs

**FIGURE 1:**
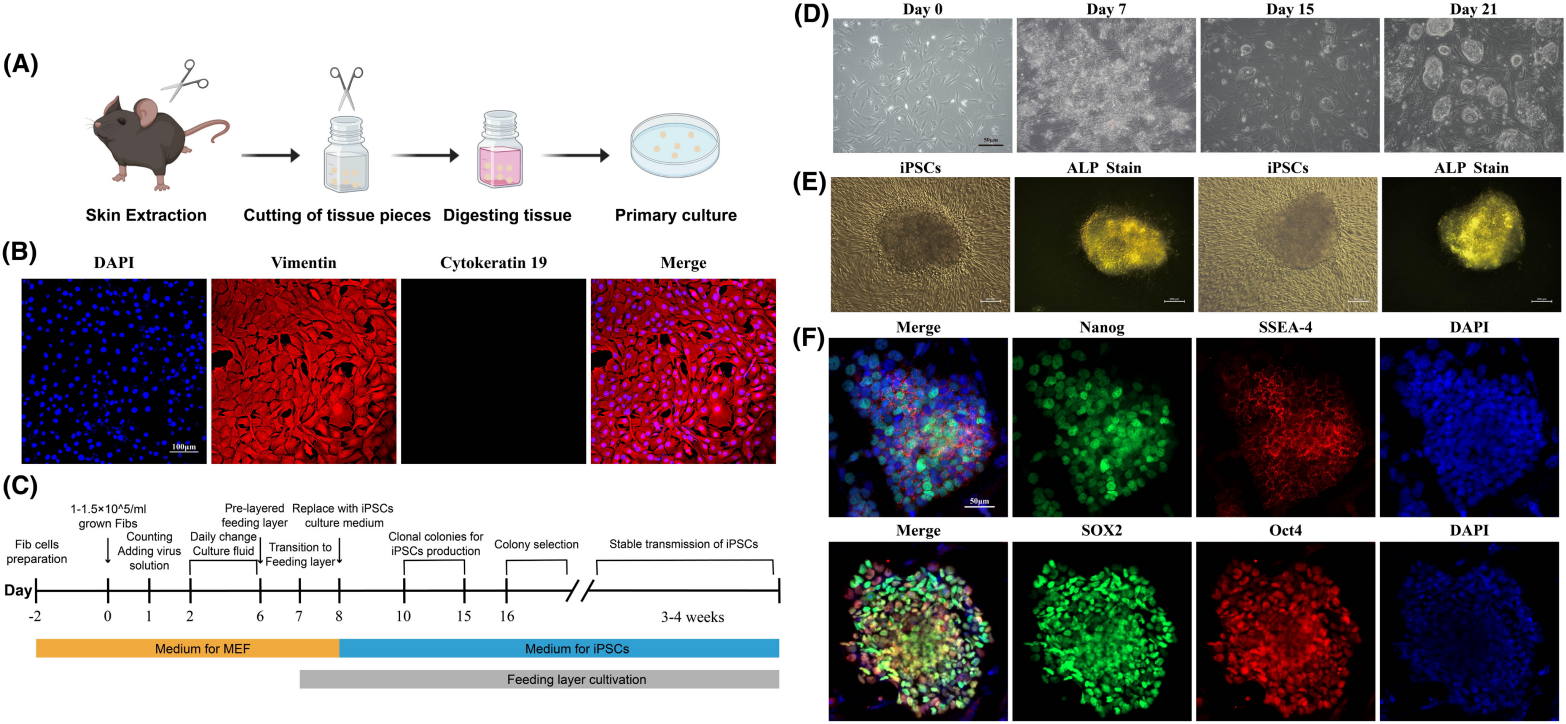
Acquisition of mouse Fibs and iPSCs construction. (A) Extraction and culture process of mouse Fibs; (B) Immunofluorescence of mouse Fibs showed high expression of vimentin and no expression of cytokeratin; (C) Flowchart of iPSCs construction; (D) Cell morphology at 0, 7, 15 and 21 d in iPSCs constructs; (E) ALP staining of iPSCs colonies showed high expression of ALP; (F) immunofluorescence of iPSCs.

Skin tissues obtained from the auricle of C57BL/6 mice were sterilized, fragmented, and digested for isolation and culture of primary Fibs (Figure 1A). Immunofluorescence results showed that the cells in the passages were typically pike‐shaped, swirled arrangement or longitudinal. The cells expressed characteristic vimentin proteins and did not express cytokeratin 19, consistent with the cellular characteristics of Fibs^25^, ^26^ (Figure 1B). The process of reprogramming mouse Fibs into iPSCs is summarized in Figure 1C. Fibs were infected with Sendai virus carrying the human transcription factors Oct4, SOX2, Klf4 and c‐Myc. Cells exhibiting mesenchymal‐epithelial transformation began to appear at 6–7 days post‐transfection, and small colony colonies appeared at approximately 12–15 days (Figure 1D). By selecting colonies and expanding them, typical iPSCs had a morphologic structure similar to that of early embryonic cells, including features such as a larger nucleus, one or several nucleoli and less cytoplasmic cytoplasm, with tightly arranged cells growing in colonies. iPSCs colony clones had a variety of morphologies, with most of them generally island‐ or nest‐shaped. After ALP live staining of iPSCs, it was observed that iPSCs colonies were stained with green fluorescence, indicating high expression of ALP in the cells (Figure 1E). Immunofluorescence staining of iPSCs for endogenous pluripotency markers (Oct4, SOX2, Nanog and SSEA‐4) demonstrated high expression of the endogenous pluripotency markers Oct4, SOX2, Nanog and SSEA‐4 within iPSCs colonies (Figure 1F). We also observed in teratoma tumorigenicity experiments in nude mice that the constructed iPSCs could differentiate into trichoblasts (Figure S1), suggesting that the constructs were successfully constructed as iPSCs via Fibs.

### 3.2 iMSCs induced and generated by iPSCs exhibit a cellular phenotype and differentiation potential similar to that of BMSCs

**FIGURE 2:**
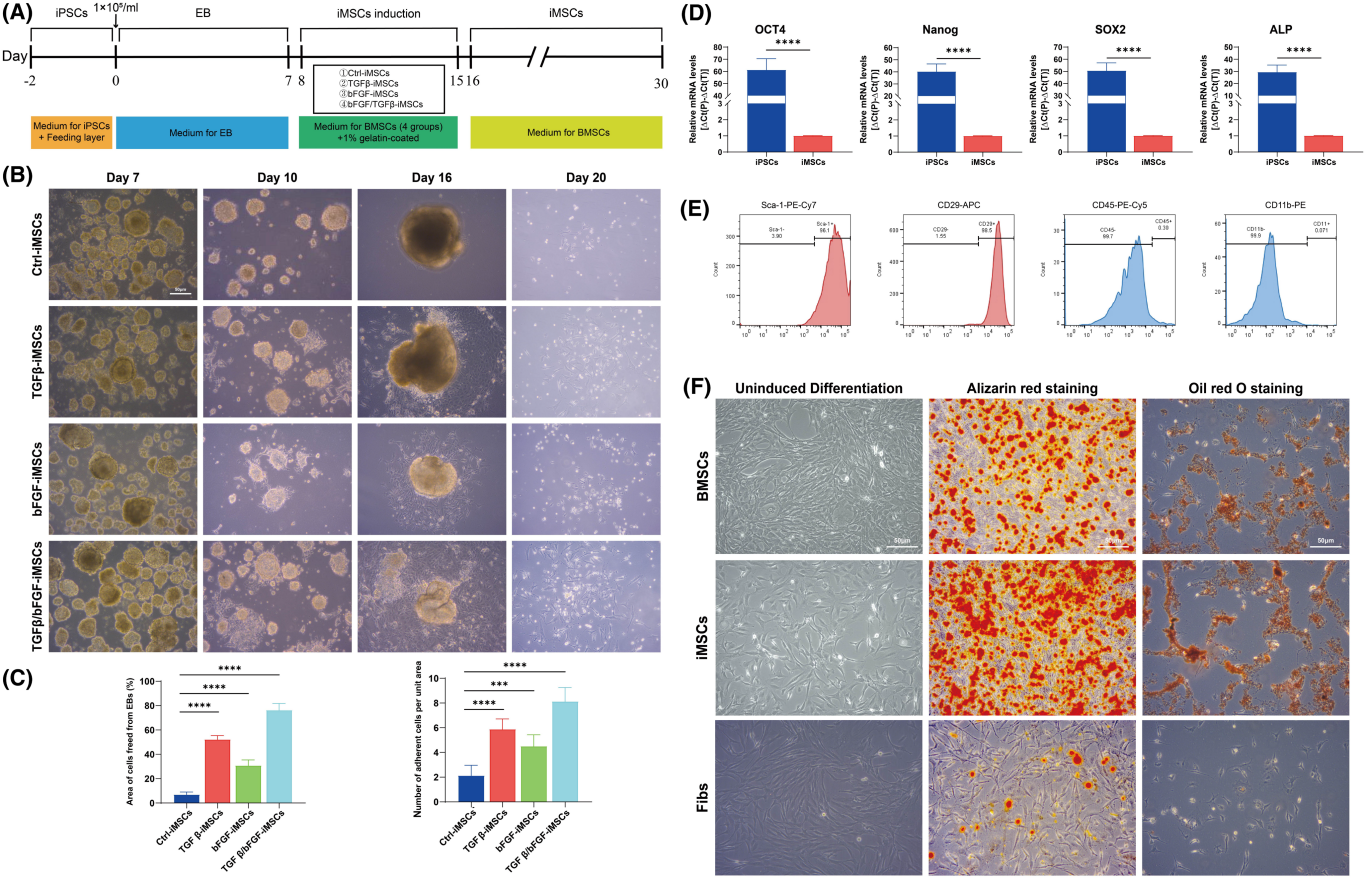
Induction and biological characterization validation of iMSCs. (A) Flow diagram of EB generation by iPSCs and induction as iMSCs; (B) Cell growth of EB induction as iMSCs; (C) EB adherent condition and percentage growth area of iMSCs showed higher induction of TGF‐β and bFGF; (D) A significant decrease in the mRNA expression of iMSCs pluripotency markers; (E) iMSCs flow results showing high expression of Sca‐1 and CD29, does not express CD45 and CD11b; (F) BMSCs and iMSCs showed numerous calcium nodules detected by Alizarin red staining after osteogenic induction, and intracellular fat particles were revealed by oil red O staining after adipogenic induction. ***p < 0.001, ****p < 0.0001.

Induction of derivation from iPSCs to iMSCs was performed according to previous research methods^27^, ^28^, ^29^ by inducing iPSCs to EB followed by further induction of derivation to iMSCs (Figure 2A). iPSCs were induced with TGF‐β or bFGF after generating EB on the 7th day. iPSCs were induced with either TGF‐β or bFGF. On the 10th day of induction, the number of wall affixations was significantly increased in the TGF‐β1 and bFGF group compared with the other groups, and a large number of iMSCs were free around the EB on the 16th day. On the 20th day, the iMSCs could be stably passaged (Figure 2B). The number of EB affixed to the wall per unit area and the number of cells around the EB were counted in each group on the 15th day. The results showed that the combined induction of TGF‐β and bFGF had a better induction effect (Figure 2C). RNA from iPSCs and iMSCs was extracted for RT–qPCR expression detection. The results showed that the mRNA expression of Oct4, Nanog, SOX2 and ALP was significantly reduced after induction (*p* < 0.0001), indicating that the pluripotency of iMSCs was decreased compared with that of iPSCs (Figure 2D). We previously validated the primary extraction and cellular characterization of mouse BMSCs^14^, ^24^ and used BMSCs as a reference to characterize the cellular properties of iMSCs using flow cytometry and osteoblastogenic lipid‐induced differentiation assays. The results showed that iMSCs highly expressed the BMSCs surface markers Sca‐1 and CD29 and did not express CD45 and CD11b, which was consistent with the mesenchymal stem cells surface marker expression characteristics (Figure 2E). After induction under certain conditions, both iMSCs and BMSCs showed similar differentiation characteristics and could successfully differentiate into osteoblasts and adipocytes (Figure 2F).

### 3.3 iMSCs exhibit faster proliferation rates and tumour‐free and BMSCs‐like transcriptome phenotypes

**FIGURE 3:**
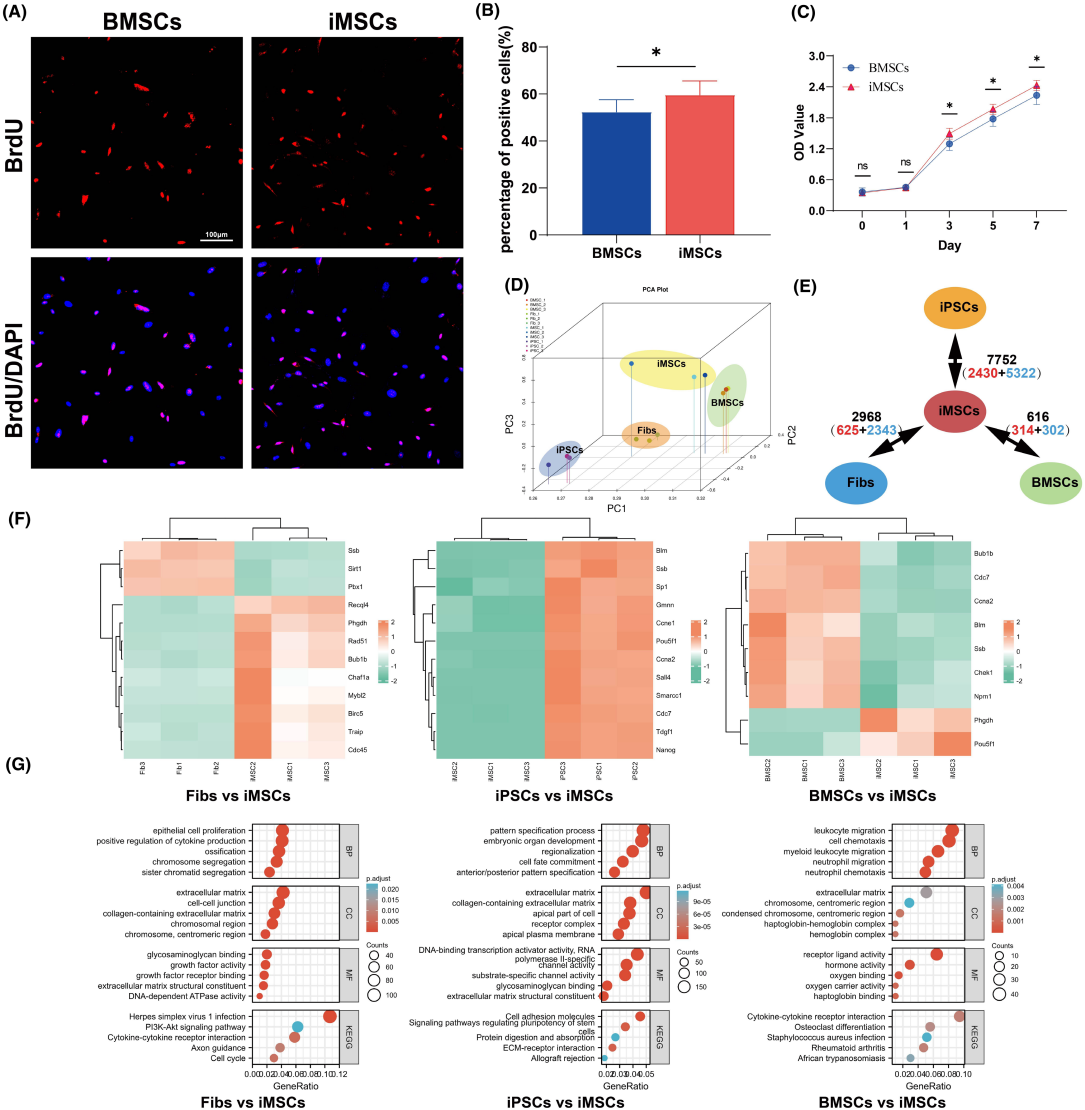
Proliferation and transcriptome sequencing analysis of iMSCs. (A) Fluorescence staining results of BMSCs and iMSCs after 48 h; (B) Statistics of the percentage of positive cells showing more iMSCs and BMSCs than BMSCs; (C) CCK‐8 proliferation rate of BMSCs and iMSCs showing a faster proliferation rate; (D) PCA principal component analysis of iMSCs, Fibs, iPSCs and BMSCs; (E) Differential genes between the four cells, with red font representing upregulated genes and blue font representing downregulated genes; (F) Different genes and Mueller plurinet genes, heatmap visualization of the expression of all intersection genes; (G) GO enrichment and KEGG enrichment analysis of differential genes between the four cells. *p < 0.05.

BrdU was used to label the cell division capacity, which was fixed, stained and blocked 48 h later and photographed and counted under a confocal microscope (Figure 3A). The results showed that iMSCs split faster than BMSCs (Figure 3B *p =* 0.0206). The proliferative potential of both cell lines was determined by CCK8 at 0, 1, 3, 5, and 7 days, and the results showed that iMSCs proliferated at a faster rate (Figure 3C). We injected iMSCs into BALB/c‐nu male nude mice, and no significant tumour formation was observed subcutaneously in mice after 6 weeks (Figure S2). Pathological sections and HE staining of the site of injected cells showed only collagen and subcutaneous structures in the area, indicating that iMSCs were not tumorigenic in the subcutis.

To clarify the differences in gene expression patterns between Fibs, BMSCs, iPSCs and iMSCs, three independent replicates of culture and transcriptome sequencing were performed on these cells. The results showed tighter clustering between iMSCs and BMSCs in PCA, while iMSCs showed greater differences in gene expression with Fibs and iPSCs (Figure 3D). Two‐by‐two comparative analysis of DEGs showed that the maximum number of differentially expressed genes between iMSCs and iPSCs was 7752, accounting for 36.3% of the 21,367 genes, and the minimum number of differentially expressed genes between iMSCs and BMSCs was 616, accounting for only 2.9% of the 21,367 genes (Figure 3E). We referred to the stem cell‐specific Mueller plurinet genes proposed by Müller et al,^30^ intersected the differential genes with Mueller plurinet genes between iMSCs and Fibs, iPSCs, and BMSCs, and found that there were 12 stem cell characterization genes with differences between iMSCs and Fibs, 12 stem cell characterization genes with differences between iMSCs and iPSCs, and 9 stem cell characterization genes with differences between iMSCs and BMSCs. Heatmapping was performed for all differentially characterized stem cell genes (Figure 3F). Meanwhile, GO enrichment and KEGG enrichment analyses were performed for the top 4000 differentially expressed genes (Figure 3G). The results showed that the differentially expressed genes between iMSCs and Fibs were mainly related to epithelial cell proliferation, extracellular matrix, glycosaminoglycan binding and the PI3K‐Akt signalling pathway. The differentially expressed genes between iMSCs and iPSCs were mainly related to the pattern specification process, extracellular matrix, DNA‐binding transcription activator activity and cell adhesion molecules. The differentially expressed genes between iMSCs and BMSCs were mainly related to leukocyte migration, extracellular matrix, receptor ligand activity and cytokine‐cytokine receptor interaction.

### 3.4 iMSCs have intraosseous chemotaxis

We stained BMSCs and iMSCs with DiD, injected them into mice via the tail vein, and collected different organs of mice at 4 h, 1, 7, and 14 days for sectioning and cytofluorescence observation. The results showed that a large number of fluorescent signals of DiD‐BMSCs and DiD‐iMSCs were present in the lungs (Figure S3A), followed by the liver (Figure S3B), spleen (Figure S3C) and femoral bone marrow (Figure S3D). Fewer aggregated organs and tissues were kidney (Figure S3E) and heart (Figure S3F), and almost no cells were deposited in brain tissue (Figure S3G). Because of the high number of cells in the lungs, liver, spleen and femoral bone marrow cavity after cell infusion and significant quantitative changes over time, quantitative fluorescence statistical analyses were performed for the above four tissues and organs (Figure S3H,I). Infused iMSCs and BMSCs appeared in large numbers in the lungs at 4 h compared to other tissues and organs, but the number of cells in the lungs gradually declined with time, reaching a minimum at the observation time point in the lungs by 14 days. Exogenous infused cells in the liver and spleen increased progressively with time, peaking on 7 days and declining on 14 days. The iMSCs and BMSCs in the femoral marrow cavity gradually increased with time, with a peak on 14 days. Comparison of the two types of cells in the same organs revealed that iMSCs were retained in the lungs less than BMSCs at 4 h postinfusion (*p* = 0.0076, Figure S3J), while the number of iMSCs in the liver was less than that of BMSCs (*p* = 0.0042, Figure S3K), and in the spleen and femur, the distribution did not differ (*p* > 0.05, Figure S3L,M).

### 3.5 iMSCs prevent osteoporotic bone loss and reduce bone fragility

**FIGURE 4:**
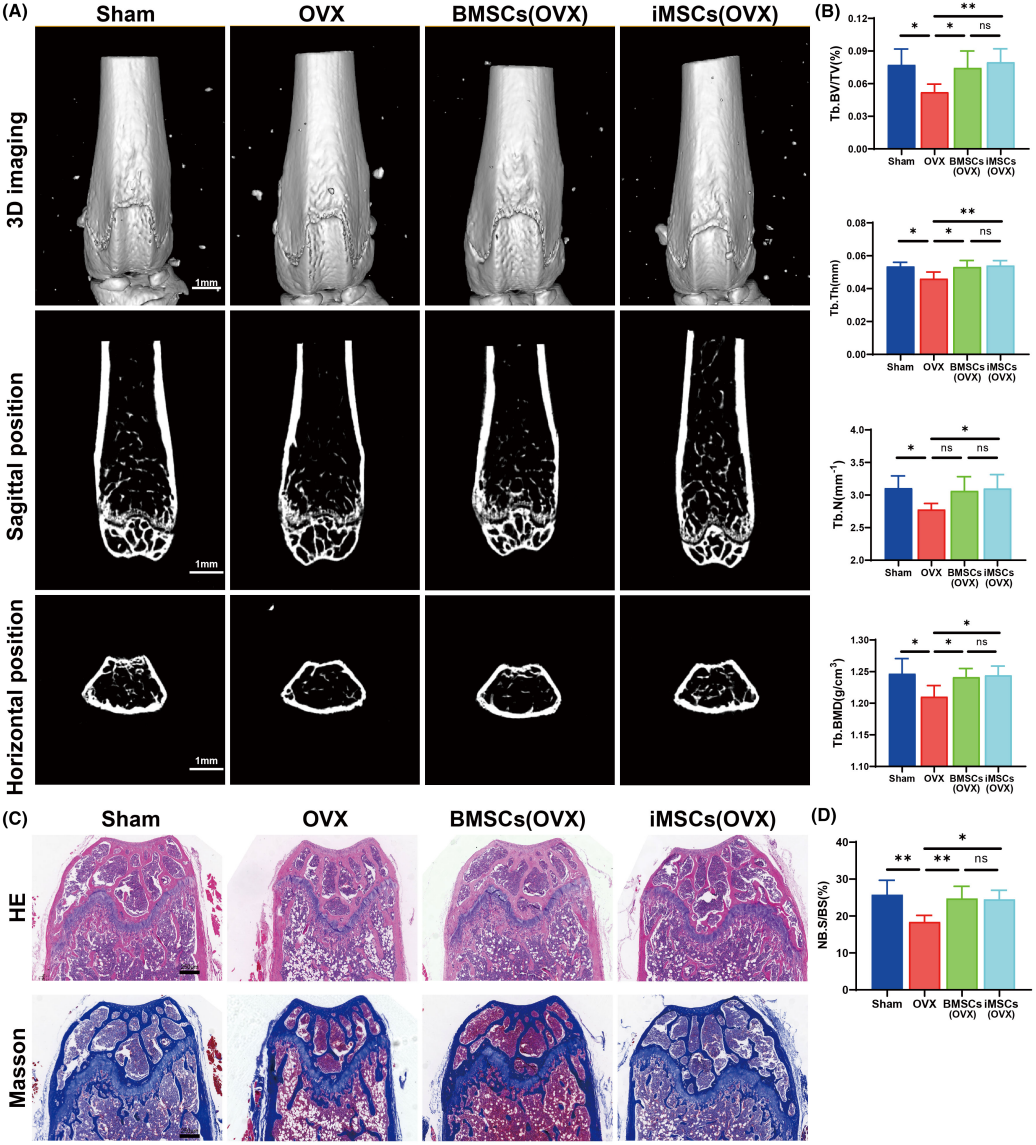
Micro‐CT and pathological histological staining of mouse femurs. (A) The results of micro CT scanning 3D reconstruction, sagittal and horizontal scanning of femur; (B) results of analysis of distal femoral bone; (C) HE staining and Masson staining results of femoral sections in each group; (D) Statistical results of new bone generation with Masson staining. *p < 0.05, **p < 0.01, ns *p* > 0.05.

We used C57BL/6 female mice to construct an ovariectomized osteoporosis model. Bone mass and bone biomechanical changes were assessed on 35 days after OVX, that is, 28 days after cell injection. The femurs of the Sham, OVX, iMSCs and BMSCs groups were scanned using microCT (Figure 4A). Semiquantitative analysis of femoral micro CT scans showed (Figure 4B) that Tb. BV/TV (*p* = 0.0149), Tb. Th (*p* = 0.0129), Tb. N (*p* = 0.029), and Tb. BMD (*p* = 0.0141) was significantly lower in femoral tissues in the OVX group than in those in the Sham group. Tb. BV/TV (*p* = 0.0074), Tb. Th (*p* = 0.008), Tb. N (*p* = 0.0314), and Tb. BMD (*p* = 0.0226) was significantly higher in the iMSCs group than in the OVX group. Tb. BV/TV (*p* = 0.0311), Tb. Th (*p* = 0.0177), and Tb. BMD (*p* = 0.0385) was significantly higher in the BMSCs group than in the OVX group, and there was no significant difference in Tb. N (*p* = 0.0617). There was no difference in Tb. BV/TV (*p* = 0.8803), Tb. Th (*p* = 0.9765), Tb. N (*p* = 0.9837), and Tb. BMD (*p* = 0.9923) in the BMSCs group compared with the iMSCs group. Biomechanical testing of mouse femurs on 35 days after OVX (Table S2) revealed that OVX resulted in a significant decrease in elastic load, breaking load and stiffness and a significant increase in maximum displacement of the femur. In contrast, compared to the OVX group, infusion of either iMSCs or BMSCs increased the elastic load, breaking load and stiffness of the femur and decreased the maximum displacement. These results suggest that iMSCs significantly reduced OVX‐induced bone loss and decreased bone fragility in cancellous bone in mice.

We performed HE staining and Masson staining after sectioning femoral tissues to assess the changes in morphology within the bone after iMSCs transplantation (Figure 4C). HE staining showed no significant morphological changes between groups. Masson staining showed that OVX resulted in reduced new bone formation near the femoral growth plate, whereas the iMSCs and BMSCs groups promoted bone and collagen maturation compared to the OVX group (iMSCs vs. OVX: *p* = 0.0127, BMSCs vs. OVX: *p* = 0.0099, Figure 4D). These results suggest that both BMSCs and iMSCs can promote new bone generation.

### 3.6 Metabolomic analysis of mouse femur and plasma

**FIGURE 5:**
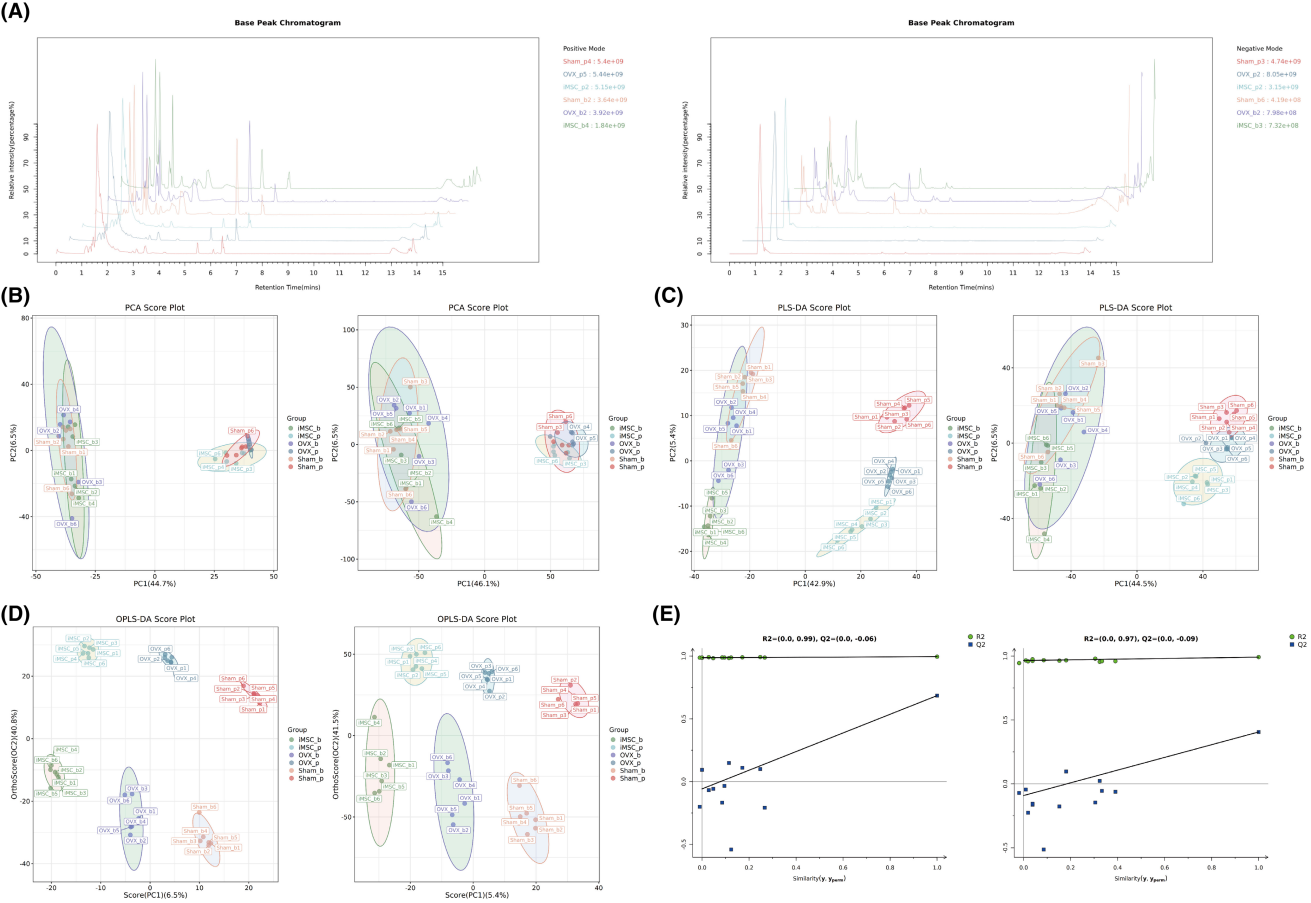
Multivariate statistical analysis of bone and plasma metabolites in mice based on LC–MS. (A) Chromatogram of base peak intensity in the Sham, OVX and iMSCs (OVX) groups in positive and negative ion modes; (B) PCA scores in positive and negative ion modes; (C) PLS‐DA scores in positive and negative ion modes; (D) OPLS‐DA scores in positive and negative ion modes; (E) PLS‐DA displacement test in positive and negative ion modes.

Six independent femoral tissue samples and plasma samples from each group were fully scanned for positive and negative ions using LC–MS under optimal conditions. To ensure the stability of instrument operation, one QC sample was interspersed with every 10 samples. The good reproducibility of LS–MS was evident from the typical basal peak intensity chromatograms of the Sham, OVX and iMSCs groups (Figure 5A). After matching, extracting and normalizing the peaks, PCA showed significant differences in the variation between groups (Figure 5B). While there is still some overlap, there is also a clear trend toward separation. The effect on metabolite patterns after cell transplantation was investigated using PLS‐DA and OPLS‐DA models. The results showed positive patterns of 0.562, 0.995 and 0.878 and negative patterns of 0.564, 0.992, and 0.751 for *R* ^2^X, *R* ^2^Y and *Q* ^2^, respectively, in the PLS‐DA model score plot (Figure 5C). The positive patterns of *R* ^2^X, *R* ^2^Y and *Q* ^2^ in the OPLS‐DA model were 0.562, 0.995 and 0.685, respectively; the negative patterns were 0.564, 0.992 and 0.406, respectively (Figure 5D). Positive pattern in PLS‐DA permutation test: *R* ^2^ = (0.0, 0.99), *Q* ^2^ = (0.0, 0.69); negative pattern: *R* ^2^ = (0.0, 0.96), *Q* ^2^ = (0.0, 0.4) (Figure 5E). The above results indicate that the PLS‐DA and OPLS‐DA models are of high quality.

### 3.7 Identification of potential intraosseous and plasma biomarkers in mice after iMSCs transplantation

Variables were selected to search for significantly altered metabolites based on VIP values in the OPLS‐DA model. To identify the metabolites that contributed most to the clustering, variables with VIP values higher than 1.0 and independent *t*‐tests of *p* < 0.05 between the two groups were selected. Metabolites were identified based on the accuracy provided by the HMDB, METLIN, LIPID MAPS and KEGG databases and validated using MS/MS fragment ion information. Finally, 15 metabolites of different abundance in bone tissue and 30 metabolites of different abundance in plasma were identified. Tables S3 and S4 list basic information on potential biomarkers screened in bone and plasma, fold change (FC) of the biologically corresponding pathway, and *p*‐value. Box plots were used to visualize the differences in potential biomarkers in bone and plasma screened between the Sham, OVX and iMSCs groups (Figure S4). The ROC curves of potential biomarkers in bone for iMSCs with anti‐osteoporotic effects were plotted based on OPLS‐DA (Figure S5). The figure shows that the biomarkers in bone and plasma have high sensitivity AUC values (>0.80), suggesting that they can be used as potential biological targets of action and biomarkers in bone for the anti‐osteoporotic effects of iMSCs infusion.

### 3.8 Biological pathways and functional analyses of metabolites

**FIGURE 6:**
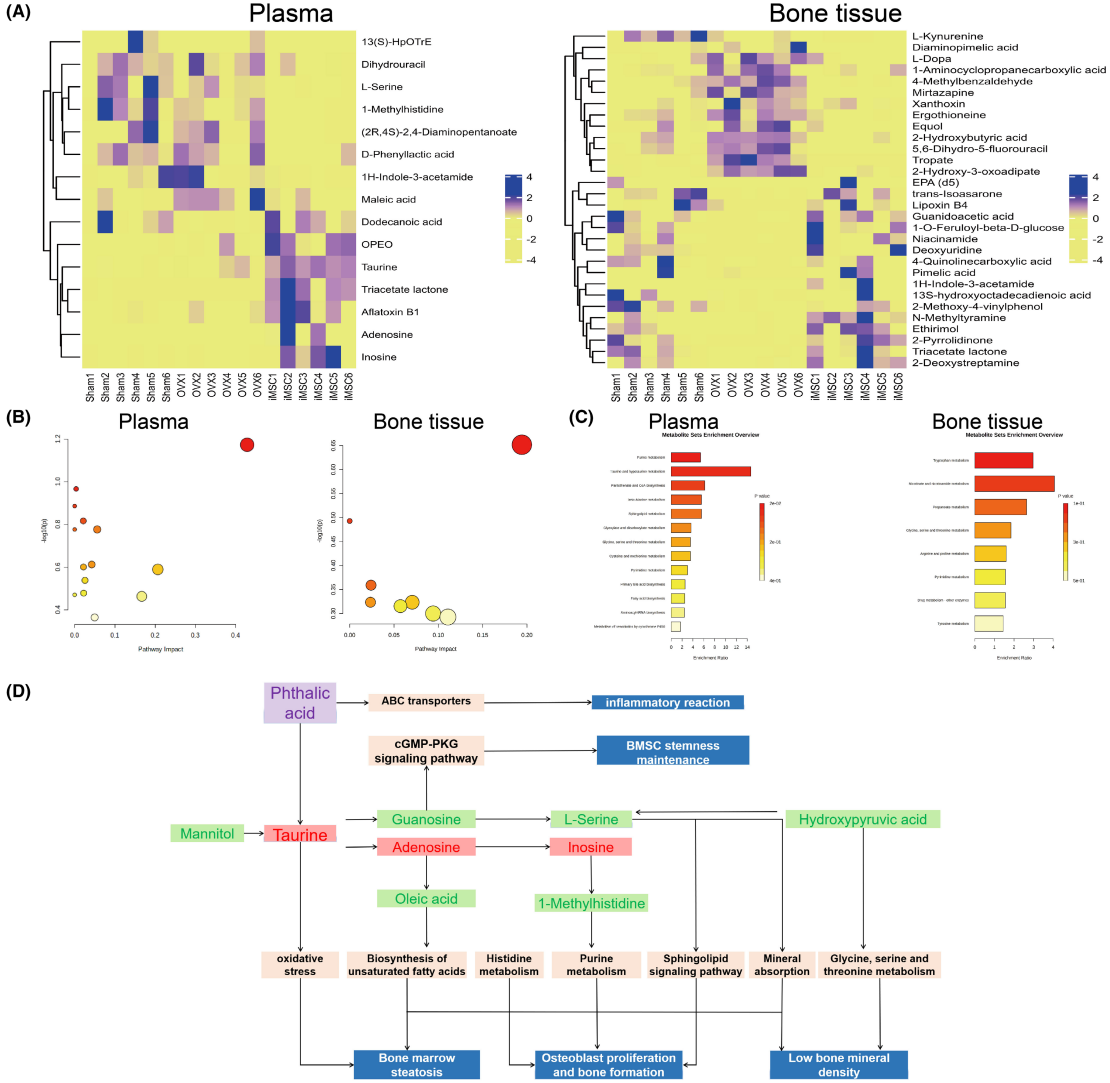
Heatmap and Metaboanalyst pathway enrichment analysis of bone tissue and plasma biomarkers. (A) Heatmap analysis of biomarkers; blue and yellow indicate increased and decreased levels, respectively; (B) Analysis of metabolic pathway impact; (C) Overview of metabolite enrichment; (D) Interaction of metabolic pathways and biological phenotype of relevant metabolites screened based on iMSCs intervention.

Heatmap analysis showed that biomarkers in the intraosseous and peripheral blood (Figure 6A) iMSCs groups were similar to those in the Sham group. In contrast, there was a significant difference between the OVX group and the iMSCs group, suggesting that iMSCs significantly improved the metabolic profile in the bone tissue of osteoporotic mice. Pathway and enrichment analyses of 30 important biomarkers in peripheral blood and 15 important biomarkers in bone tissue were performed according to Metaboanalyst 5.0. After analysing and visualizing the relevant metabolic pathways, calculations based on pathway topology analysis identified taurine and taurine metabolism, purine metabolism and histidine metabolism as the three metabolic pathways with the most important impacts after intraosseous iMSCs infusion (Table S5) and nicotinic acid and nicotinamide metabolism, propionic acid metabolism, and glycine/serine/threonine metabolism as the three most important metabolic pathways with the most important impacts in peripheral blood (Table S6). Metabolite enrichment analyses showed that taurine and subtaurine metabolism, purine metabolism, histidine metabolism, pantothenate, and coenzyme A biosynthesis, and sphingolipid metabolism were the top five metabolite enrichment sets altered by iMSCs in bone (Figure 6C). In peripheral blood nicotinic acid and nicotinamide metabolism, propionic acid metabolism, glycine, serine and threonine metabolism, arginine and proline metabolism, and drug metabolism‐other enzymes were the top five metabolite condensed sets altered by iMSCs (Figure 6C). The above results suggest that the metabolism of amino acids and lipids is a key factor involved in bone protection and the prevention of osteoporosis formation in iMSCs. Through database searches (KEGG) and a review of relevant literature,^31^, ^32^, ^33^, ^34^ we found that these metabolites are mainly associated with purine metabolism, histidine metabolism and lipid metabolism but also with oxidative stress and inflammatory responses. These metabolic pathways are usually closely associated with bone formation and bone resorption (Figure 6D). By mapping the metabolic pathways of these significantly different metabolic markers, we can more intuitively reflect the relationship between these metabolites and the osteoporosis phenotype.

## 4 DISCUSSION

Postmenopausal osteoporosis is one of the most common types of osteoporosis and is characterized by low BMD due to abnormal bone metabolism.^35^, ^36^ The main clinical treatments for osteoporosis are exercise interventions and medication, but long‐term medication use can lead to serious side effects.^37^ Stem cells and their related products have made great strides in treating osteoporosis.^37^, ^38^, ^39^, ^40^, ^41^, ^42^, ^43^ However, there are still risks of immune rejection and tumorigenesis in stem cell therapy, and efficacy is closely related to donor age, cell status and number of generations of expansion.^15^ The use of iPSCs induction to generate iMSCs has the property of unlimited growth and differentiation, which avoids the ethical controversy associated with embryonic stem cells being the source of embryos and can be a potential alternative source of BMSCs.^16^, ^44^, ^45^, ^46^

iMSCs are a class of derived stem cells with strong and reliable regenerative capacity. Over the past decade, several protocols have been developed to generate iMSCs from iPSCs for induction. The earliest induction of iMSCs from embryonic stem cells, such as deprivation of the feeder layer or nonadhesive encapsulation, was used, and the resulting cells had an immunophenotype similar to that of BMSCs.^47^, ^48^, ^49^ Later, researchers found that the addition of bFGF resulted in iMSCs from iPSCs, which were highly similar to mesenchymal stem cells in terms of morphology and marker expression.^50^, ^51^, ^52^ Whereas some of the studies have looked at signal regulation in terms of mimicking embryonic development, the mesoderm of the EB is thought to be the main source of mesenchymal stem cells, which develop into adipose and skeletal tissue.^53^, ^54^ The TGF‐β signalling pathway is closely associated with embryonic development, epithelial mesenchymalization and the development and maintenance of various organs in stem cells.^55^, ^56^ Therefore, the use of TGF‐β as an inducer was also effective in maintaining iMSCs transdifferentiation.^27^, ^28^ In the present study, based on the summary of iMSCs induction methods,^27^ iPSCs were induced into EB, and then iMSCs with stable transmission, morphology and phenotype in accordance with BMSCs were generated under the induction of TGF‐β and bFGF cytokines. iMSCs were also closer to BMSCs in terms of their gene expression patterns, and iMSCs did not show similar tumorigenicity to iPSCs. The results further indicated that the coinduction of TGF‐β and bFGF was more efficient than that of a single cytokine, and the coinduction effectively promoted the freeing and expansion of iMSCs from EB.

In this study, we employed tail vein infusion to investigate the distribution pattern and anti‐osteoporotic effects of iMSCs in ovariectomized mice. We found that after intravenous injection, a large number of iMSCs accumulated in the lungs, followed by the liver, spleen and femoral marrow cavity, which is consistent with previous reports.^57^, ^58^ The initial capture of cells in the lungs reflects the well‐known “pulmonary first‐pass effect” in stem cell delivery, aligning with earlier studies.^58^ However, we observed a low level of iMSC accumulation in the kidneys and heart, and almost no deposition in the brain tissue, suggesting a low affinity of these tissues for iMSCs. Furthermore, 4 h after iMSC infusion, the retention of iMSCs in the lungs and liver was lower than that of BMSCs, while their distribution in the spleen and femur showed no significant differences, possibly due to differences in tissue affinity and cell diameter between iMSCs and BMSCs. Notably, the iMSCs demonstrated a higher affinity for the femoral marrow cavity, reaching a peak at Day 14, which facilitates their recruitment into bone tissue to participate in bone repair.^59^ Over time, the number of iMSCs in the femoral marrow gradually increased, indicating that bone tissue has a specific chemotactic attraction, which is crucial for bone regeneration. This suggests that iMSCs have the potential to become a targeted therapy for osteoporosis, as they not only survive but also actively home back to the bone marrow to promote bone formation. This finding is particularly valuable for the treatment of osteoporosis, as it underscores the ability of iMSCs to migrate to bone tissue after systemic administration. Such homing capability could reduce the need for localized injections, offering a less invasive option for long‐term osteoporosis treatment. The use of iMSCs could be optimized to enable targeted delivery to bone tissue in subsequent therapies, thereby enhancing the efficacy of osteoporosis treatment while reducing systemic side effects.

The mechanisms by which stem cells inhibit bone resorption and promote bone formation are more complex. Differences in the energy metabolism and antioxidant defence systems of mesenchymal stem cells from different sources have been found to affect the efficacy of cell therapy in osteoporosis treatment.^60^ Stem cells can also be involved in systemic immunomodulation, angiogenesis and inflammatory responses systemically or in bone by secreting soluble paracrine factors, such as TGF‐β, prostaglandin E2 and vascular endothelial growth factor.^14^, ^61^, ^62^, ^63^ Additionally, MSCs play a key role in regulating the bone microenvironment through immunomodulation. MSCs interact with various immune cells, such as macrophages, T cells and dendritic cells, to modulate the inflammatory environment.^64^, ^65^ By promoting the polarization of macrophages from the pro‐inflammatory M1 phenotype to the anti‐inflammatory M2 phenotype, MSCs help reduce inflammation, which is a key factor exacerbating bone loss in osteoporosis patients.^65^ This immunomodulation is crucial for creating a favourable environment for bone repair and regeneration. Furthermore, MSCs secrete a variety of cytokines and growth factors, including transforming growth factor‐beta (TGF‐β), interleukin‐10 (IL‐10), and vascular endothelial growth factor (VEGF), which promote osteoblast activity and inhibit osteoclast‐mediated bone resorption.^38^, ^66^, ^67^ In osteoporosis, excessive osteoclast activity leads to increased bone degradation, but MSC‐derived cytokines help restore the dynamic balance between osteoclasts and osteoblasts. The interaction between MSCs and osteoclasts is particularly important in osteoporosis, where bone resorption exceeds bone formation. Through their paracrine signalling, MSCs can inhibit the differentiation and activity of osteoclasts, thereby alleviating the excessive bone loss characteristic of osteoporosis.^68^ This regulatory effect on osteoclasts highlights the therapeutic potential of MSCs, as they not only stimulate bone formation but also control bone resorption, addressing both aspects of the pathological imbalance in osteoporosis.^69^ In summary, MSCs have the ability to differentiate into osteoblasts, regulate immune responses and modulate osteoclast activity, thus playing a multifaceted role in bone regeneration. These functions make MSCs a potential target for treating osteoporosis, offering prospects for bone tissue regeneration and restoring bone balance. In the present study, we found that iMSCs transplantation into ovariectomized mice was able to increase the elastic load, breaking load and stiffness of the femur and decrease the maximum displacement. The microCT results demonstrated that iMSCs transplantation resulted in an elevation of Tb. BV/TV, Tb. N, Tb. BMD, Ct. BMD, Ct. BV and Ct. Th in the femur of the mouse, which was in line with previous reports.^70^ This finding is consistent with the pro‐osteogenic effect of BMSCs that we verified.^14^ Transplantation of iMSCs was able to promote collagen maturation within the bone. This suggests that exogenous iMSCs infusion may mediate the osteogenesis in bone, acting as a significant osteogenic and potentially anti‐fracture agent.

Intrabony metabolic disorders are important predisposing mechanisms for osteoporosis, and metabolomics can be effective in assessing osteoporosis efficacy and finding new therapeutic targets. Untargeted and targeted metabolomics have been widely used in osteoporosis mechanism and treatment studies, especially drug studies.^71^, ^72^, ^73^ In this study, LC–MS was used for metabolite changes in the femur and peripheral blood of mice after iMSCs infusion. Fifteen key metabolites, including L‐serine, dihydrouracil, and taurine, were screened in bone tissue, and 30 key metabolites, including 2‐pyrrolidone, 1‐aminocyclopropanecarboxylic acid and 2‐hydroxybutyric acid, were screened in peripheral blood. Of these, lactone triacetate, 1H‐indole‐3‐acetamide and maleic acid were common differential biomarkers in plasma and bone. Previous studies have identified a number of potential biomarkers of osteoporosis associated with osteoporosis,^74^, ^75^, ^76^, ^77^ and the present study further enriches the database of potential markers of osteoporosis and provides additional guidance for the diagnosis of osteoporosis. Meanwhile, we found that the effects of iMSCs infusion on the metabolic pathways in osteoporotic bone were mainly related to taurine and taurine metabolism, purine metabolism, histidine metabolism etc., and the effects on the metabolic pathways in peripheral blood were mainly related to nicotinic acid and nicotinamide metabolism, propionic acid metabolism, glycine/serine/threonine metabolism, etc., and the result was partially in agreement with other metabolites found in the serum.^78^, ^79^ More validation of these potential metabolic pathway modulations is necessary in the future to elucidate the potential mechanism of action of stem cell therapy for osteoporosis.

Overall, the iMSCs we obtained promoted OVX‐induced bone structural remodelling in osteoporotic mice, were able to influence a variety of key metabolite changes in intraosseous and peripheral blood, and had potential anti‐osteoporotic fracture effects. However, we acknowledge that our study is insufficient. First, this study did not explore the role of human‐derived iMSCs in a mouse model given the immune rejection caused by species differences, and future comparisons between murine and human‐derived iMSCs are needed. Second, the in vivo distribution of iMSCs was not studied in all organs and tissues throughout the body, and cell tracer techniques need to be summarized for a more precise study of the dynamic in vivo distribution of iMSCs. Meanwhile, the bone tissue of mice lacks Haver's system, which may differ from humans in the formation of osteoporosis. The anti‐osteoporotic effects and metabolic mechanisms of iMSCs in all bone tissues of the whole body in other large animal models need to be further investigated.

## 5 CONCLUSIONS

The iMSCs derived from fibroblasts in this study exhibit biological characteristics similar to those of BMSCs. Following intravenous infusion, iMSCs accumulate predominantly in the lungs and gradually increase in the femoral bone marrow cavity over time. Importantly, iMSCs promote the remodelling of bone structure in OVX‐induced osteoporotic mice, demonstrating potential anti‐osteoporotic and anti‐fracture effects. Additionally, iMSC infusion influences several key metabolites in both bone and peripheral blood, some of which may serve as potential biomarkers and therapeutic targets for iMSC‐based interventions in osteoporosis. In summary, this study explores the application of iMSCs in osteoporosis treatment and lays an important foundation for translational research on the use of transdifferentiated stem cells in osteoporosis therapy.

## AUTHOR CONTRIBUTIONS

**Wei‐Zhou Wang:** Conceptualization (equal); data curation (equal); formal analysis (equal); funding acquisition (equal); methodology (equal); resources (equal); software (equal); visualization (equal); writing – original draft (equal); writing – review and editing (equal). **Yang‐Hao Wang:** Conceptualization (equal); data curation (equal); formal analysis (equal); funding acquisition (equal); methodology (equal); resources (equal); software (equal); visualization (equal); writing – original draft (equal); writing – review and editing (equal). **Fei He:** Conceptualization (equal); funding acquisition (equal); methodology (equal); supervision (equal); writing – review and editing (equal). **Sha‐Sha Bao:** Data curation (equal); methodology (equal); software (equal); visualization (equal); writing – review and editing (equal). **Guoyu Li:** Data curation (equal); methodology (equal); software (equal); writing – review and editing (equal). **Guang Yang:** Data curation (equal); methodology (equal); software (equal); writing – review and editing (equal). **Jing Chen:** Data curation (equal); methodology (equal); software (equal); writing – review and editing (equal). **Xin‐Yu Yang:** Data curation (equal); methodology (equal); software (equal); writing – review and editing (equal). **Ya Xiao:** Data curation (equal); methodology (equal); software (equal); writing – review and editing (equal). **Ya‐Shuang Tong:** Data curation (equal); methodology (equal); visualization (equal); writing – review and editing (equal). **Xue‐Ting Zhao:** Data curation (equal); methodology (equal); software (equal); writing – review and editing (equal). **Jun Hu:** Data curation (equal); formal analysis (equal); funding acquisition (equal); methodology (equal); software (equal); writing – original draft (equal); writing – review and editing (equal). **Ding‐You You:** Data curation (equal); methodology (equal); supervision (equal); writing – review and editing (equal).

## FUNDING INFORMATION

This study was supported by the National Natural Science Foundation of China (82460428, 82073569), Yunnan Province Department of Science and Technology‐Kunming Medical University Joint Special Project (202201AY070001‐057; 202301AY070001‐268), Biomedical Specialization of Yunnan Province (202402AA310012), the Innovative Research Team of Yunnan Province (202405AS350016), Yunnan Provincial Department of Science and Technology Basic Research Program Youth Project (202401AU070046), Yunnan Provincial Department of Education Scientific Research Fund Teacher Category Project‐Young Talent Basic Research Special Fund (2024 J0180), Kunming Medical University Postgraduate Innovation Fund(2024B005) and Kunming Medical University Student Innovation and Entrepreneurship Training Program Project (202210678018, 2023JXD032, 2023JXD088).

## CONFLICT OF INTEREST STATEMENT

The author(s) declared no potential conflicts of interest concerning the research, authorship, and/or publication of this article.

## Supporting information


Data S1.

## Data Availability

The datasets supporting the conclusions of this article are available by contacting the corresponding author (Email: drhefei@126.com). The Supplementary Material for this article can be found online. All sequencing data have been uploaded to the GEO database with the accession number Series GSE222324 (https://www.ncbi.nlm.nih.gov/geo/query/acc.cgi?acc=GSE222324). The raw data can be reviewed after entering the secure token (cbonquusjxctdsd) in the GEO database.

## References

[1] Suzman R , Beard JR , Boerma T , Chatterji S . Health in an ageing world—what do we know? Lancet. 2015;385(9967):484‐486. 25468156 10.1016/S0140-6736(14)61597-X

[2] Johnell O , Kanis JA . An estimate of the worldwide prevalence and disability associated with osteoporotic fractures. Osteoporos Int. 2006;17(12):1726‐1733. 16983459 10.1007/s00198-006-0172-4

[3] Kanis JA , McCloskey EV , Johansson H , Oden A , Melton LJ 3rd , Khaltaev N . A reference standard for the description of osteoporosis. Bone. 2008;42(3):467‐475. 18180210 10.1016/j.bone.2007.11.001

[4] Ayub N , Faraj M , Ghatan S , Reijers JAA , Napoli N , Oei L . The treatment gap in osteoporosis. J Clin Med. 2021;10(13):3002. 34279485 10.3390/jcm10133002 PMC8268346

[5] Kim DY , Ko SH . Common regulators of lipid metabolism and bone marrow adiposity in postmenopausal women. Pharmaceuticals (Basel). 2023;16(2):322. 10.3390/ph16020322 PMC9967016 37259464

[6] Takahashi A , Yousif A , Hong L , Chefetz I . Premature ovarian insufficiency: pathogenesis and therapeutic potential of mesenchymal stem cell. J Mol Med (Berl). 2021;99(5):637‐650. 33641066 10.1007/s00109-021-02055-5

[7] An F , Wang X , Wang C , et al. Research progress on the role of lncRNA‐miRNA networks in regulating adipogenic and osteogenic differentiation of bone marrow mesenchymal stem cells in osteoporosis. Front Endocrinol (Lausanne). 2023;14:1210627. 37645421 10.3389/fendo.2023.1210627 PMC10461560

[8] Kiernan J , Hu S , Grynpas MD , Davies JE , Stanford WL . Systemic mesenchymal stromal cell transplantation prevents functional bone loss in a mouse model of age‐related osteoporosis. Stem Cells Transl Med. 2016;5(5):683‐693. 26987353 10.5966/sctm.2015-0231 PMC4835247

[9] Fu YS , Lu CH , Chu KA , et al. Xenograft of human umbilical mesenchymal stem cells from Wharton's jelly differentiating into osteocytes and reducing osteoclast activity reverses osteoporosis in ovariectomized rats. Cell Transplant. 2018;27(1):194‐208. 29562774 10.1177/0963689717750666 PMC6434481

[10] Kong F , Shi X , Xiao F , et al. Transplantation of hepatocyte growth factor‐modified dental pulp stem cells prevents bone loss in the early phase of ovariectomy‐induced osteoporosis. Hum Gene Ther. 2018;29(2):271‐282. 28950723 10.1089/hum.2017.091

[11] Yu Z , Zhu T , Li C , et al. Improvement of intertrochanteric bone quality in osteoporotic female rats after injection of polylactic acid‐polyglycolic acid copolymer/collagen type I microspheres combined with bone mesenchymal stem cells. Int Orthop. 2012;36(10):2163‐2171. 22539160 10.1007/s00264-012-1543-4 PMC3460074

[12] Yu Y , Shao B , Zhou Z , et al. Role of bone marrow‐derived mesenchymal stem cells in treating estrogen deficiency induced osteoporosis. Xi Bao Yu Fen Zi Mian Yi Xue Za Zhi. 2013;29(12):1267‐1271. 24321070

[13] Cao L , Liu G , Gan Y , et al. The use of autologous enriched bone marrow MSCs to enhance osteoporotic bone defect repair in long‐term estrogen deficient goats. Biomaterials. 2012;33(20):5076‐5084. 22504017 10.1016/j.biomaterials.2012.03.069

[14] Wang W , Wang Y , Tang Z , et al. Mesenchymal stem cells prevent ovariectomy‐induced osteoporosis formation in mice through intraosseous vascular remodeling. Biochem Biophys Res Commun. 2021;582:64‐71. 34689107 10.1016/j.bbrc.2021.10.033

[15] Stolzing A , Jones E , McGonagle D , Scutt A . Age‐related changes in human bone marrow‐derived mesenchymal stem cells: consequences for cell therapies. Mech Ageing Dev. 2008;129(3):163‐173. 18241911 10.1016/j.mad.2007.12.002

[16] Zhao C , Ikeya M . Generation and applications of induced pluripotent stem cell‐derived mesenchymal stem cells. Stem Cells Int. 2018;2018:9601623. 30154868 10.1155/2018/9601623 PMC6091255

[17] Kim H , Zhao Q , Barreda H , et al. Identification of molecules responsible for therapeutic effects of extracellular vesicles produced from iPSC‐derived MSCs on Sjogren's syndrome. Aging Dis. 2021;12(6):1409‐1422. 34527418 10.14336/AD.2021.0621 PMC8407887

[18] Liang X , Lin F , Ding Y , et al. Conditioned medium from induced pluripotent stem cell‐derived mesenchymal stem cells accelerates cutaneous wound healing through enhanced angiogenesis. Stem Cell Res Ther. 2021;12(1):295. 34016178 10.1186/s13287-021-02366-x PMC8139053

[19] Zhou M , Xi J , Cheng Y , et al. Reprogrammed mesenchymal stem cells derived from iPSCs promote bone repair in steroid‐associated osteonecrosis of the femoral head. Stem Cell Res Ther. 2021;12(1):175. 33712030 10.1186/s13287-021-02249-1 PMC7953570

[20] Jungbluth P , Spitzhorn LS , Grassmann J , et al. Human iPSC‐derived iMSCs improve bone regeneration in mini‐pigs. Bone Res. 2019;7:32. 31667001 10.1038/s41413-019-0069-4 PMC6813363

[21] Teo GS , Yang Z , Carman CV , Karp JM , Lin CP . Intravital imaging of mesenchymal stem cell trafficking and association with platelets and neutrophils. Stem Cells. 2015;33(1):265‐277. 25263183 10.1002/stem.1848 PMC4270897

[22] Sipkins DA , Wei X , Wu JW , et al. In vivo imaging of specialized bone marrow endothelial microdomains for tumour engraftment. Nature. 2005;435(7044):969‐973. 15959517 10.1038/nature03703 PMC2570168

[23] Lo Celso C , Fleming HE , Wu JW , et al. Live‐animal tracking of individual haematopoietic stem/progenitor cells in their niche. Nature. 2009;457(7225):92‐96. 19052546 10.1038/nature07434 PMC2820276

[24] Wang W , Wang Y , Hu J , et al. Untargeted metabolomics reveal the protective effect of bone marrow mesenchymal stem cell transplantation against ovariectomy‐induced osteoporosis in mice. Cell Transplant. 2022;31:9636897221079745. 35225020 10.1177/09636897221079745 PMC8891838

[25] Schachtschneider KM , Liu Y , Mäkeläinen S , et al. Oncopig soft‐tissue sarcomas recapitulate key transcriptional features of human sarcomas. Sci Rep. 2017;7(1):2624. 28572589 10.1038/s41598-017-02912-9 PMC5453942

[26] Fullár A , Karászi K , Hollósi P , et al. Two ways of epigenetic silencing of TFPI2 in cervical cancer. PLoS One. 2020;15(6):e0234873. 32559232 10.1371/journal.pone.0234873 PMC7304613

[27] Lim SW , Kim KW , Kim BM , et al. Alleviation of renal ischemia/reperfusion injury by exosomes from induced pluripotent stem cell‐derived mesenchymal stem cells. Korean J Intern Med. 2022;37(2):411‐424. 34521186 10.3904/kjim.2020.438 PMC8925954

[28] Sheyn D , Ben‐David S , Shapiro G , et al. Human induced pluripotent stem cells differentiate into functional mesenchymal stem cells and repair bone defects. Stem Cells Transl Med. 2016;5(11):1447‐1460. 27400789 10.5966/sctm.2015-0311 PMC5070500

[29] Ishiy FA , Fanganiello RD , Griesi‐Oliveira K , et al. Improvement of in vitro osteogenic potential through differentiation of induced pluripotent stem cells from human exfoliated dental tissue towards mesenchymal‐like stem cells. Stem Cells Int. 2015;2015:249098. 25802529 10.1155/2015/249098 PMC4329829

[30] Müller FJ , Laurent LC , Kostka D , et al. Regulatory networks define phenotypic classes of human stem cell lines. Nature. 2008;455(7211):401‐405. 18724358 10.1038/nature07213 PMC2637443

[31] Wong JC , Fiscus RR . Essential roles of the nitric oxide (no)/cGMP/protein kinase G type‐Iα (PKG‐Iα) signaling pathway and the atrial natriuretic peptide (ANP)/cGMP/PKG‐Iα autocrine loop in promoting proliferation and cell survival of OP9 bone marrow stromal cells. J Cell Biochem. 2011;112(3):829‐839. 21328456 10.1002/jcb.22981

[32] Abshirini M , Ilesanmi‐Oyelere BL , Kruger MC . Potential modulatory mechanisms of action by long‐chain polyunsaturated fatty acids on bone cell and chondrocyte metabolism. Prog Lipid Res. 2021;83:101113. 34217732 10.1016/j.plipres.2021.101113

[33] Fitzpatrick LA , Buzas E , Gagne TJ , et al. Targeted deletion of histidine decarboxylase gene in mice increases bone formation and protects against ovariectomy‐induced bone loss. Proc Natl Acad Sci USA. 2003;100(10):6027‐6032. 12716972 10.1073/pnas.0934373100 PMC156320

[34] Yang K , Li J , Tao L . Purine metabolism in the development of osteoporosis. Biomed Pharmacother. 2022;155:113784. 36271563 10.1016/j.biopha.2022.113784

[35] Zou Z , Liu W , Cao L , et al. Advances in the occurrence and biotherapy of osteoporosis. Biochem Soc Trans. 2020;48(4):1623‐1636. 32627832 10.1042/BST20200005

[36] Chandra A , Rajawat J . Skeletal aging and osteoporosis: mechanisms and therapeutics. Int J Mol Sci. 2021;22(7):3553. 33805567 10.3390/ijms22073553 PMC8037620

[37] Chen T , Yang T , Zhang W , Shao J . The therapeutic potential of mesenchymal stem cells in treating osteoporosis. Biol Res. 2021;54(1):42. 34930472 10.1186/s40659-021-00366-y PMC8686520

[38] Zheng X , Wang W , Chen S , Zuo B , Li J . Transplanted mesenchymal stromal cells are unable to migrate to the bone surface and subsequently improve osteogenesis in glucocorticoid‐induced osteoporosis. Cytotherapy. 2023;25(5):472‐482. 36863932 10.1016/j.jcyt.2023.01.004

[39] Leguy D , Magro L , Pierache A , et al. Changes in bone mineral density after allogenic stem cell transplantation. Joint Bone Spine. 2022;89(5):105373. 35259477 10.1016/j.jbspin.2022.105373

[40] Lin L , He E , Wang H , et al. Intravenous transplantation of human hair follicle‐derived mesenchymal stem cells ameliorates trabecular bone loss in osteoporotic mice. Front Cell Dev Biol. 2022;10:814949. 35359450 10.3389/fcell.2022.814949 PMC8960386

[41] Yang J , Fatima K , Zhou X , He C . Meticulously engineered three‐dimensional‐printed scaffold with microarchitecture and controlled peptide release for enhanced bone regeneration. Biomater Transl. 2024;5(1):69‐83. 39220663 10.12336/biomatertransl.2024.01.007 PMC11362348

[42] Liu H , Wu Y , Wang F , et al. Bone‐targeted engineered bacterial extracellular vesicles delivering miRNA to treat osteoporosis. Compos Part B Eng. 2023;267:111047.

[43] Liu H , Zhang H , Wang SC , et al. Bone‐targeted bioengineered bacterial extracellular vesicles delivering siRNA to ameliorate osteoporosis. Compos Pt B‐Eng. 2023;225:12.

[44] Yu J , Vodyanik MA , Smuga‐Otto K , et al. Induced pluripotent stem cell lines derived from human somatic cells. Science. 2007;318(5858):1917‐1920. 18029452 10.1126/science.1151526

[45] Eberle I , Moslem M , Henschler R , Cantz T . Engineered MSCs from patient‐specific iPS cells. Adv Biochem Eng Biotechnol. 2013;130:1‐17. 22915200 10.1007/10_2012_156

[46] Lian Q , Zhang Y , Zhang J , et al. Functional mesenchymal stem cells derived from human induced pluripotent stem cells attenuate limb ischemia in mice. Circulation. 2010;121(9):1113‐1123. 20176987 10.1161/CIRCULATIONAHA.109.898312

[47] Olivier EN , Rybicki AC , Bouhassira EE . Differentiation of human embryonic stem cells into bipotent mesenchymal stem cells. Stem Cells. 2006;24(8):1914‐1922. 16644919 10.1634/stemcells.2005-0648

[48] Hwang NS , Varghese S , Lee HJ , et al. In vivo commitment and functional tissue regeneration using human embryonic stem cell‐derived mesenchymal cells. Proc Natl Acad Sci USA. 2008;105(52):20641‐20646. 19095799 10.1073/pnas.0809680106 PMC2634917

[49] Hynes K , Menicanin D , Mrozik K , Gronthos S , Bartold PM . Generation of functional mesenchymal stem cells from different induced pluripotent stem cell lines. Stem Cells Dev. 2014;23(10):1084‐1096. 24367908 10.1089/scd.2013.0111 PMC4015475

[50] Boyd NL , Robbins KR , Dhara SK , West FD , Stice SL . Human embryonic stem cell‐derived mesoderm‐like epithelium transitions to mesenchymal progenitor cells. Tissue Eng Part A. 2009;15(8):1897‐1907. 19196144 10.1089/ten.tea.2008.0351 PMC2792108

[51] Lian Q , Zhang Y , Liang X , Gao F , Tse HF . Directed differentiation of human‐induced pluripotent stem cells to mesenchymal stem cells. Methods Mol Biol. 2016;1416:289‐298. 27236679 10.1007/978-1-4939-3584-0_17

[52] Karlsson C , Emanuelsson K , Wessberg F , et al. Human embryonic stem cell‐derived mesenchymal progenitors—potential in regenerative medicine. Stem Cell Res. 2009;3(1):39‐50. 19515621 10.1016/j.scr.2009.05.002

[53] Sánchez L , Gutierrez‐Aranda I , Ligero G , et al. Enrichment of human ESC‐derived multipotent mesenchymal stem cells with immunosuppressive and anti‐inflammatory properties capable to protect against experimental inflammatory bowel disease. Stem Cells. 2011;29(2):251‐262. 21732483 10.1002/stem.569

[54] Mahmood A , Harkness L , Schrøder HD , Abdallah BM , Kassem M . Enhanced differentiation of human embryonic stem cells to mesenchymal progenitors by inhibition of TGF‐beta/activin/nodal signaling using SB‐431542. J Bone Miner Res. 2010;25(6):1216‐1233. 20200949 10.1002/jbmr.34

[55] Watabe T , Miyazono K . Roles of TGF‐beta family signaling in stem cell renewal and differentiation. Cell Res. 2009;19(1):103‐115. 19114993 10.1038/cr.2008.323

[56] Kahata K , Dadras MS , Moustakas A . TGF‐β Family Signaling in epithelial differentiation and epithelial‐mesenchymal transition. Cold Spring Harb Perspect Biol. 2018;10(1):a022194. 28246184 10.1101/cshperspect.a022194 PMC5749157

[57] Kidd S , Spaeth E , Dembinski JL , et al. Direct evidence of mesenchymal stem cell tropism for tumor and wounding microenvironments using in vivo bioluminescent imaging. Stem Cells. 2009;27(10):2614‐2623. 19650040 10.1002/stem.187 PMC4160730

[58] Fischer UM , Harting MT , Jimenez F , et al. Pulmonary passage is a major obstacle for intravenous stem cell delivery: the pulmonary first‐pass effect. Stem Cells Dev. 2009;18(5):683‐692. 19099374 10.1089/scd.2008.0253 PMC3190292

[59] Huang S , Xu L , Sun Y , Zhang Y , Li G . The fate of systemically administrated allogeneic mesenchymal stem cells in mouse femoral fracture healing. Stem Cell Res Ther. 2015;6:206. 26503505 10.1186/s13287-015-0198-7 PMC4621860

[60] Zheng CX , Sui BD , Liu N , et al. Adipose mesenchymal stem cells from osteoporotic donors preserve functionality and modulate systemic inflammatory microenvironment in osteoporotic cytotherapy. Sci Rep. 2018;8(1):5215. 29581449 10.1038/s41598-018-23098-8 PMC5980002

[61] Larrick JW , Mendelsohn AR . Mesenchymal stem cells for frailty? Rejuvenation Res. 2017;20(6):525‐529. 29179649 10.1089/rej.2017.2042

[62] Yeo GEC , Ng MH , Nordin FB , Law JX . Potential of mesenchymal stem cells in the rejuvenation of the aging immune system. Int J Mol Sci. 2021;22(11):5749. 34072224 10.3390/ijms22115749 PMC8198707

[63] Hofer HR , Tuan RS . Secreted trophic factors of mesenchymal stem cells support neurovascular and musculoskeletal therapies. Stem Cell Res Ther. 2016;7(1):131. 27612948 10.1186/s13287-016-0394-0 PMC5016979

[64] Özdemir R , Özdemir AT , Sarıboyacı AE , Uysal O , Tuğlu M , Kırmaz C . The investigation of immunomodulatory effects of adipose tissue mesenchymal stem cell educated macrophages on the CD4 T cells. Immunobiology. 2019;224(4):585‐594. 31072631 10.1016/j.imbio.2019.04.002 PMC7124282

[65] Hu K , Shang Z , Yang X , Zhang Y , Cao L . Macrophage polarization and the regulation of bone immunity in bone homeostasis. J Inflamm Res. 2023;16:3563‐3580. 37636272 10.2147/JIR.S423819 PMC10460180

[66] Takano T , Li YJ , Kukita A , et al. Mesenchymal stem cells markedly suppress inflammatory bone destruction in rats with adjuvant‐induced arthritis. Lab Investig. 2014;94(3):286‐296. 24395111 10.1038/labinvest.2013.152

[67] Guo D , Yang J , Liu D , Zhang P , Sun H , Wang J . Human umbilical cord mesenchymal stem cells overexpressing RUNX1 promote tendon‐bone healing by inhibiting osteolysis, enhancing osteogenesis and promoting angiogenesis. Genes Genomics. 2024;46(4):461‐473. 38180714 10.1007/s13258-023-01478-3

[68] Jiménez‐Ortega RF , Ortega‐Meléndez AI , Patiño N , Rivera‐Paredez B , Hidalgo‐Bravo A , Velázquez‐Cruz R . The involvement of microRNAs in bone remodeling signaling pathways and their role in the development of osteoporosis. Biology. 2024;13(7):505. 39056698 10.3390/biology13070505 PMC11273958

[69] Jiang Y , Zhang P , Zhang X , Lv L , Zhou Y . Advances in mesenchymal stem cell transplantation for the treatment of osteoporosis. Cell Prolif. 2021;54(1):e12956. 33210341 10.1111/cpr.12956 PMC7791182

[70] Sui BD , Chen J , Zhang XY , et al. Gender‐independent efficacy of mesenchymal stem cell therapy in sex hormone‐deficient bone loss via immunosuppression and resident stem cell recovery. Exp Mol Med. 2018;50(12):1‐14. 10.1038/s12276-018-0192-0 PMC6297134 30559383

[71] Reck G , Kronitz B , Breckwoldt M . Significance of the estriol profile as an endogenous function test of the fetoplacental unit. Geburtshilfe Frauenheilkd. 1987;47(11):774‐780. 3452325 10.1055/s-2008-1036044

[72] Qin W , Gao J , Yan J , et al. Microarray analysis of signalling interactions between inflammation and angiogenesis in subchondral bone in temporomandibular joint osteoarthritis. Biomater Transl. 2024;5(2):175‐184. 39351165 10.12336/biomatertransl.2024.02.007 PMC11438608

[73] Lewis JW , Frost K , Neag G , et al. Therapeutic avenues in bone repair: harnessing an anabolic osteopeptide, PEPITEM, to boost bone growth and prevent bone loss. Cell Rep Med. 2024;5(5):101574. 38776873 10.1016/j.xcrm.2024.101574 PMC11148860

[74] Kou J , He C , Cui L , et al. Discovery of potential biomarkers for postmenopausal osteoporosis based on untargeted GC/LC‐MS. Front Endocrinol (Lausanne). 2022;13:849076. 35518930 10.3389/fendo.2022.849076 PMC9062097

[75] Lau KT , Krishnamoorthy S , Sing CW , Cheung CL . Metabolomics of osteoporosis in humans: a systematic review. Curr Osteoporos Rep. 2023;21(3):278‐288. 37060383 10.1007/s11914-023-00785-8

[76] Li Y , Si Y , Ma Y , Yin H . Application and prospect of metabolomics in the early diagnosis of osteoporosis: a narrative review. Bioanalysis. 2023;15(22):1369‐1379. 37695026 10.4155/bio-2023-0131

[77] Li X , Wang ZY , Ren N , et al. Identifying therapeutic biomarkers of zoledronic acid by metabolomics. Front Pharmacol. 2023;14:1084453. 37180703 10.3389/fphar.2023.1084453 PMC10166846

[78] Jiang YC , Li YF , Zhou L , Zhang DP . UPLC‐MS metabolomics method provides valuable insights into the effect and underlying mechanisms of Rhizoma Drynariae protecting osteoporosis. J Chromatogr B Analyt Technol Biomed Life Sci. 2020;1152:122262. 10.1016/j.jchromb.2020.122262 32682315

[79] Zhao JF , Xu JY , Xu YE , et al. High‐throughput metabolomics method for discovering metabolic biomarkers and pathways to reveal effects and molecular mechanism of ethanol extract from Epimedium against osteoporosis. Front Pharmacol. 2020;11:1318. 32973531 10.3389/fphar.2020.01318 PMC7481463

