# Supplementary material for: iPSCs‐derived iMSCs prevent osteoporotic bone loss and affect bone metabolites in ovariectomized mice
Source: J Cell Mol Med. 2024 Nov 24;28(22):e70200. doi: 10.1111/jcmm.70200 (PMC11586054; doi:10.1111/jcmm.70200)
Supplement: Supplementary file 1 — Data S1. [file JCMM-28-e70200-s001.zip › jcmm70200-sup-0006-Supplementary Table 1.docx]

**Supplementary Table 1. Primers for the target genes used for RT-qPCR**

| Primer | Sequence(5’-3’) | Product length(bp) |
| --- | --- | --- |
| OCT4-F | AGGAAGCCGACAACAATGAGAACC | 386 |
| OCT4-R | AGGGACTGAGTAGAGTGTGGTGAAG |  |
| NANOG-F | AGTACCTCAGCCTCCAGCAGATG | 220 |
| NANOG-R | CCAGATGCGTTCACCAGATAGCC |  |
| SOX2-F | GGTTACCTCTTCCTCCCACTCCAG | 221 |
| SOX2-R | TCCCCTTCTCCAGTTCGCAGTC |  |
| ALP-F | CGGCGTCCATGAGCAGAACTAC | 302 |
| ALP-R | CCAGATACAGGCAAGGCAGATAGC |  |
