# Supplementary material for: iPSCs‐derived iMSCs prevent osteoporotic bone loss and affect bone metabolites in ovariectomized mice
Source: J Cell Mol Med. 2024 Nov 24;28(22):e70200. doi: 10.1111/jcmm.70200 (PMC11586054; doi:10.1111/jcmm.70200)
Supplement: Supplementary file 1 — Data S1. [file JCMM-28-e70200-s001.zip › jcmm70200-sup-0007-Supplementary Table 2.docx]

**Supplementary Table 2. Results of testing the mechanical properties of the mouse femur**

| designation | unit | **Sham** | **OVX** | **BMSC** | **iMSC** |
| --- | --- | --- | --- | --- | --- |
| Elastic load | N | 14.71 ± 4.88^**^ | 9.02 ± 3.12 | 13.51 ± 3.51^**^ | 13.75 ± 4.05^**^ |
| maximum displacement | mm | 0.18 ± 0.03^*^ | 0.26 ± 0.03 | 0.20 ± 0.04^*^ | 0.19 ± 0.04^*^ |
| crippling load | N | 18.04 ± 3.63^**^ | 12.97 ± 4.24 | 17.82 ± 3.96^**^ | 16.77 ± 3.05^**^ |
| stiffness | N/mm | 83.25 ± 15.12^**^ | 60.67 ± 16.32 | 71.83 ± 14.71^*^ | 76.92 ± 13.66^**^ |

Note: All results are presented as mean ± SD, and all groups were compared to the OVX group, where * *P* <0.05 and * * *P* <0.01.
