# Supplementary material for: iPSCs‐derived iMSCs prevent osteoporotic bone loss and affect bone metabolites in ovariectomized mice
Source: J Cell Mol Med. 2024 Nov 24;28(22):e70200. doi: 10.1111/jcmm.70200 (PMC11586054; doi:10.1111/jcmm.70200)
Supplement: Supplementary file 1 — Data S1. [file JCMM-28-e70200-s001.zip › jcmm70200-sup-0010-Supplementary Table 5.docx]

**Supplementary Table 5. 15 biomarkers metabolic pathway analysis**

| **Pathway Name** | **Match Status** | **p** | **-log(p)** | **Holm p** | **FDR** | **Impact** | **Details** |
| --- | --- | --- | --- | --- | --- | --- | --- |
| [Taurine and hypotaurine metabolism](https://www.metaboanalyst.ca/MetaboAnalyst/Secure/pathway/ResultView.xhtml) | [1/8](https://www.metaboanalyst.ca/MetaboAnalyst/Secure/pathway/ResultView.xhtml) | 0.067158 | 1.1729 | 1.0 | 1.0 | 0.42857 | [KEGG](http://www.genome.jp/kegg-bin/show_pathway?mmu00430) [SMP](http://www.smpdb.ca/view/SMP63675) |
| [Purine metabolism](https://www.metaboanalyst.ca/MetaboAnalyst/Secure/pathway/ResultView.xhtml) | [2/66](https://www.metaboanalyst.ca/MetaboAnalyst/Secure/pathway/ResultView.xhtml) | 0.10814 | 0.96603 | 1.0 | 1.0 | 0.00366 | [KEGG](http://www.genome.jp/kegg-bin/show_pathway?mmu00230) [SMP](http://www.smpdb.ca/view/SMP63668) |
| [Histidine metabolism](https://www.metaboanalyst.ca/MetaboAnalyst/Secure/pathway/ResultView.xhtml) | [1/16](https://www.metaboanalyst.ca/MetaboAnalyst/Secure/pathway/ResultView.xhtml) | 0.13013 | 0.88562 | 1.0 | 1.0 | 0.0 | [KEGG](http://www.genome.jp/kegg-bin/show_pathway?mmu00340) [SMP](http://www.smpdb.ca/view/SMP63632) |
| [Pantothenate and CoA biosynthesis](https://www.metaboanalyst.ca/MetaboAnalyst/Secure/pathway/ResultView.xhtml) | [1/19](https://www.metaboanalyst.ca/MetaboAnalyst/Secure/pathway/ResultView.xhtml) | 0.15272 | 0.81612 | 1.0 | 1.0 | 0.02143 | [KEGG](http://www.genome.jp/kegg-bin/show_pathway?mmu00770) [SMP](http://www.smpdb.ca/view/SMP63647) |
| [Sphingolipid metabolism](https://www.metaboanalyst.ca/MetaboAnalyst/Secure/pathway/ResultView.xhtml) | [1/21](https://www.metaboanalyst.ca/MetaboAnalyst/Secure/pathway/ResultView.xhtml) | 0.16747 | 0.77606 | 1.0 | 1.0 | 0.0 | [KEGG](http://www.genome.jp/kegg-bin/show_pathway?mmu00600) [SMP](http://www.smpdb.ca/view/SMP63667) |
| [beta-Alanine metabolism](https://www.metaboanalyst.ca/MetaboAnalyst/Secure/pathway/ResultView.xhtml) | [1/21](https://www.metaboanalyst.ca/MetaboAnalyst/Secure/pathway/ResultView.xhtml) | 0.16747 | 0.77606 | 1.0 | 1.0 | 0.05597 | [KEGG](http://www.genome.jp/kegg-bin/show_pathway?mmu00410) [SMP](http://www.smpdb.ca/view/SMP63599) |
| [Glyoxylate and dicarboxylate metabolism](https://www.metaboanalyst.ca/MetaboAnalyst/Secure/pathway/ResultView.xhtml) | [1/32](https://www.metaboanalyst.ca/MetaboAnalyst/Secure/pathway/ResultView.xhtml) | 0.24447 | 0.61177 | 1.0 | 1.0 | 0.04233 | [KEGG](http://www.genome.jp/kegg-bin/show_pathway?mmu00630) |
| [Cysteine and methionine metabolism](https://www.metaboanalyst.ca/MetaboAnalyst/Secure/pathway/ResultView.xhtml) | [1/33](https://www.metaboanalyst.ca/MetaboAnalyst/Secure/pathway/ResultView.xhtml) | 0.25113 | 0.6001 | 1.0 | 1.0 | 0.02184 | [KEGG](http://www.genome.jp/kegg-bin/show_pathway?mmu00270) [SMP](http://www.smpdb.ca/view/SMP63607) [SMP](http://www.smpdb.ca/view/SMP63637) |
| [Glycine, serine and threonine metabolism](https://www.metaboanalyst.ca/MetaboAnalyst/Secure/pathway/ResultView.xhtml) | [1/34](https://www.metaboanalyst.ca/MetaboAnalyst/Secure/pathway/ResultView.xhtml) | 0.25774 | 0.58881 | 1.0 | 1.0 | 0.20661 | [KEGG](http://www.genome.jp/kegg-bin/show_pathway?mmu00260) [SMP](http://www.smpdb.ca/view/SMP63626) |
| [Pyrimidine metabolism](https://www.metaboanalyst.ca/MetaboAnalyst/Secure/pathway/ResultView.xhtml) | [1/39](https://www.metaboanalyst.ca/MetaboAnalyst/Secure/pathway/ResultView.xhtml) | 0.28999 | 0.53762 | 1.0 | 1.0 | 0.0256 | [KEGG](http://www.genome.jp/kegg-bin/show_pathway?mmu00240) [SMP](http://www.smpdb.ca/view/SMP63658) |
| [Primary bile acid biosynthesis](https://www.metaboanalyst.ca/MetaboAnalyst/Secure/pathway/ResultView.xhtml) | [1/46](https://www.metaboanalyst.ca/MetaboAnalyst/Secure/pathway/ResultView.xhtml) | 0.33296 | 0.4776 | 1.0 | 1.0 | 0.02239 | [KEGG](http://www.genome.jp/kegg-bin/show_pathway?mmu00120) [SMP](http://www.smpdb.ca/view/SMP63601) |
| [Fatty acid biosynthesis](https://www.metaboanalyst.ca/MetaboAnalyst/Secure/pathway/ResultView.xhtml) | [1/47](https://www.metaboanalyst.ca/MetaboAnalyst/Secure/pathway/ResultView.xhtml) | 0.3389 | 0.46993 | 1.0 | 1.0 | 0.0 | [KEGG](http://www.genome.jp/kegg-bin/show_pathway?mmu00061) [SMP](http://www.smpdb.ca/view/SMP63613) |
| [Aminoacyl-tRNA biosynthesis](https://www.metaboanalyst.ca/MetaboAnalyst/Secure/pathway/ResultView.xhtml) | [1/48](https://www.metaboanalyst.ca/MetaboAnalyst/Secure/pathway/ResultView.xhtml) | 0.34479 | 0.46244 | 1.0 | 1.0 | 0.16667 | [KEGG](http://www.genome.jp/kegg-bin/show_pathway?mmu00970) |
| [Metabolism of xenobiotics by cytochrome P450](https://www.metaboanalyst.ca/MetaboAnalyst/Secure/pathway/ResultView.xhtml) | [1/64](https://www.metaboanalyst.ca/MetaboAnalyst/Secure/pathway/ResultView.xhtml) | 0.43269 | 0.36382 | 1.0 | 1.0 | 0.04965 | [KEGG](http://www.genome.jp/kegg-bin/show_pathway?mmu00980) |
