# Supplementary material for: iPSCs‐derived iMSCs prevent osteoporotic bone loss and affect bone metabolites in ovariectomized mice
Source: J Cell Mol Med. 2024 Nov 24;28(22):e70200. doi: 10.1111/jcmm.70200 (PMC11586054; doi:10.1111/jcmm.70200)
Supplement: Supplementary file 1 — Data S1. [file JCMM-28-e70200-s001.zip › jcmm70200-sup-0011-Supplementary Table 6.docx]

**Supplementary Table 6. 30 Metabolic Pathway Analysis of Biomarkers**

| **Pathway Name** | **Match Status** | **p** | **-log(p)** | **Holm p** | **FDR** | **Impact** | **Details** |
| --- | --- | --- | --- | --- | --- | --- | --- |
| [Nicotinate and nicotinamide metabolism](https://www.metaboanalyst.ca/MetaboAnalyst/Secure/pathway/ResultView.xhtml) | [1/15](https://www.metaboanalyst.ca/MetaboAnalyst/Secure/pathway/ResultView.xhtml) | 0.22297 | 0.65175 | 1.0 | 1.0 | 0.1943 | [KEGG](http://www.genome.jp/kegg-bin/show_pathway?mmu00760) [SMP](http://www.smpdb.ca/view/SMP63643) |
| [Propanoate metabolism](https://www.metaboanalyst.ca/MetaboAnalyst/Secure/pathway/ResultView.xhtml) | [1/23](https://www.metaboanalyst.ca/MetaboAnalyst/Secure/pathway/ResultView.xhtml) | 0.3215 | 0.49281 | 1.0 | 1.0 | 0.0 | [KEGG](http://www.genome.jp/kegg-bin/show_pathway?mmu00640) [SMP](http://www.smpdb.ca/view/SMP63656) |
| [Glycine, serine and threonine metabolism](https://www.metaboanalyst.ca/MetaboAnalyst/Secure/pathway/ResultView.xhtml) | [1/34](https://www.metaboanalyst.ca/MetaboAnalyst/Secure/pathway/ResultView.xhtml) | 0.43759 | 0.35893 | 1.0 | 1.0 | 0.02408 | [KEGG](http://www.genome.jp/kegg-bin/show_pathway?mmu00260) [SMP](http://www.smpdb.ca/view/SMP63626) |
| [Arginine and proline metabolism](https://www.metaboanalyst.ca/MetaboAnalyst/Secure/pathway/ResultView.xhtml) | [1/38](https://www.metaboanalyst.ca/MetaboAnalyst/Secure/pathway/ResultView.xhtml) | 0.47487 | 0.32342 | 1.0 | 1.0 | 0.02346 | [KEGG](http://www.genome.jp/kegg-bin/show_pathway?mmu00330) [SMP](http://www.smpdb.ca/view/SMP63596) |
| [Drug metabolism - other enzymes](https://www.metaboanalyst.ca/MetaboAnalyst/Secure/pathway/ResultView.xhtml) | [1/38](https://www.metaboanalyst.ca/MetaboAnalyst/Secure/pathway/ResultView.xhtml) | 0.47487 | 0.32342 | 1.0 | 1.0 | 0.07059 | [KEGG](http://www.genome.jp/kegg-bin/show_pathway?mmu00983) |
| [Pyrimidine metabolism](https://www.metaboanalyst.ca/MetaboAnalyst/Secure/pathway/ResultView.xhtml) | [1/39](https://www.metaboanalyst.ca/MetaboAnalyst/Secure/pathway/ResultView.xhtml) | 0.48382 | 0.31532 | 1.0 | 1.0 | 0.05729 | [KEGG](http://www.genome.jp/kegg-bin/show_pathway?mmu00240) [SMP](http://www.smpdb.ca/view/SMP63658) |
| [Tryptophan metabolism](https://www.metaboanalyst.ca/MetaboAnalyst/Secure/pathway/ResultView.xhtml) | [1/41](https://www.metaboanalyst.ca/MetaboAnalyst/Secure/pathway/ResultView.xhtml) | 0.50127 | 0.29993 | 1.0 | 1.0 | 0.09417 | [KEGG](http://www.genome.jp/kegg-bin/show_pathway?mmu00380) [SMP](http://www.smpdb.ca/view/SMP63693) |
| [Tyrosine metabolism](https://www.metaboanalyst.ca/MetaboAnalyst/Secure/pathway/ResultView.xhtml) | [1/42](https://www.metaboanalyst.ca/MetaboAnalyst/Secure/pathway/ResultView.xhtml) | 0.50978 | 0.29262 | 1.0 | 1.0 | 0.11085 | [KEGG](http://www.genome.jp/kegg-bin/show_pathway?mmu00350) [SMP](http://www.smpdb.ca/view/SMP63684) |
