# Supplementary figures and images for: iPSCs‐derived iMSCs prevent osteoporotic bone loss and affect bone metabolites in ovariectomized mice
Source: J Cell Mol Med. 2024 Nov 24;28(22):e70200. doi: 10.1111/jcmm.70200 (PMC11586054; doi:10.1111/jcmm.70200)

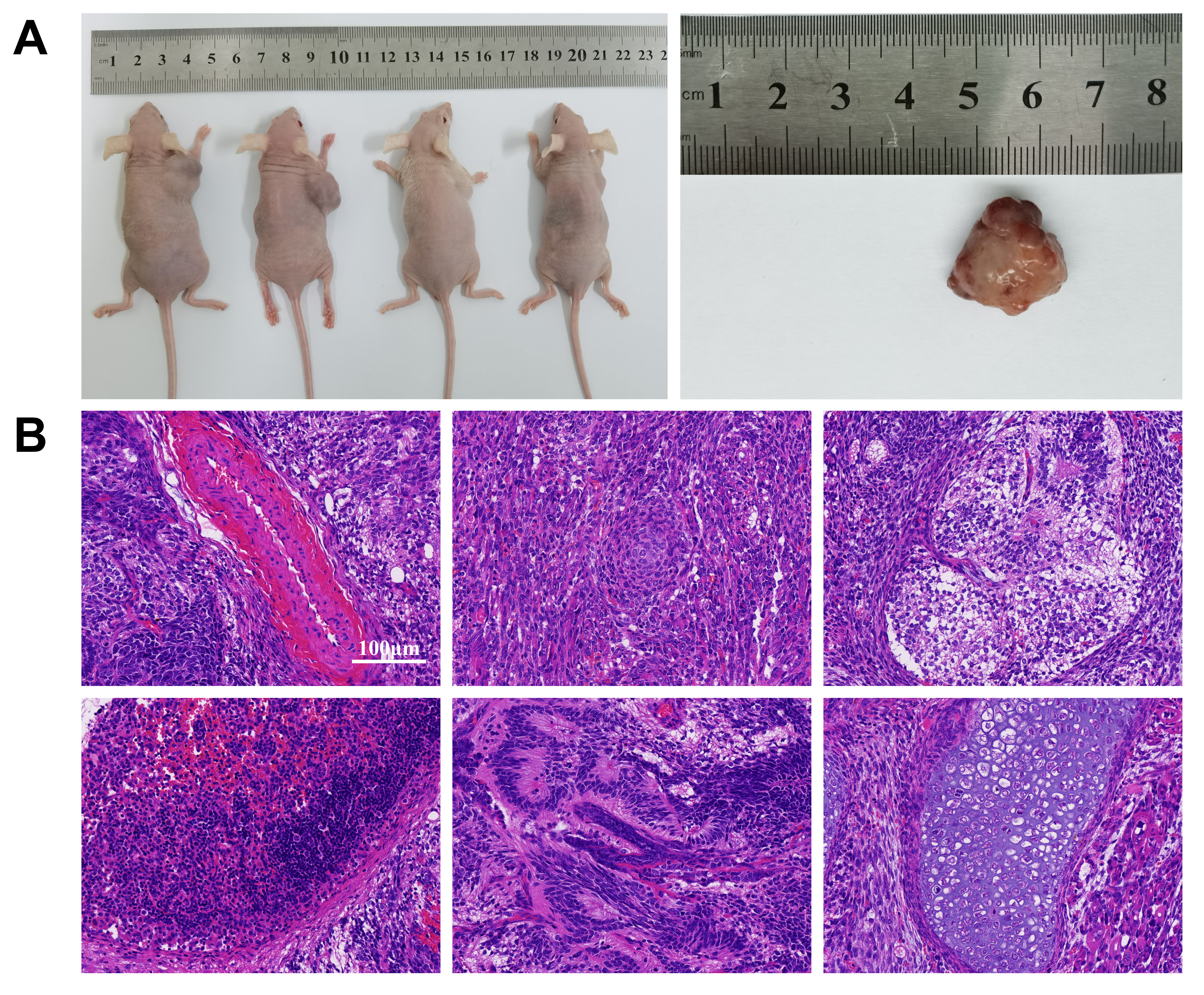

Supplement: Supplementary file 1 — Data S1. [file JCMM-28-e70200-s001.zip › jcmm70200-sup-0001-Supplementary Figure 1.jpg]

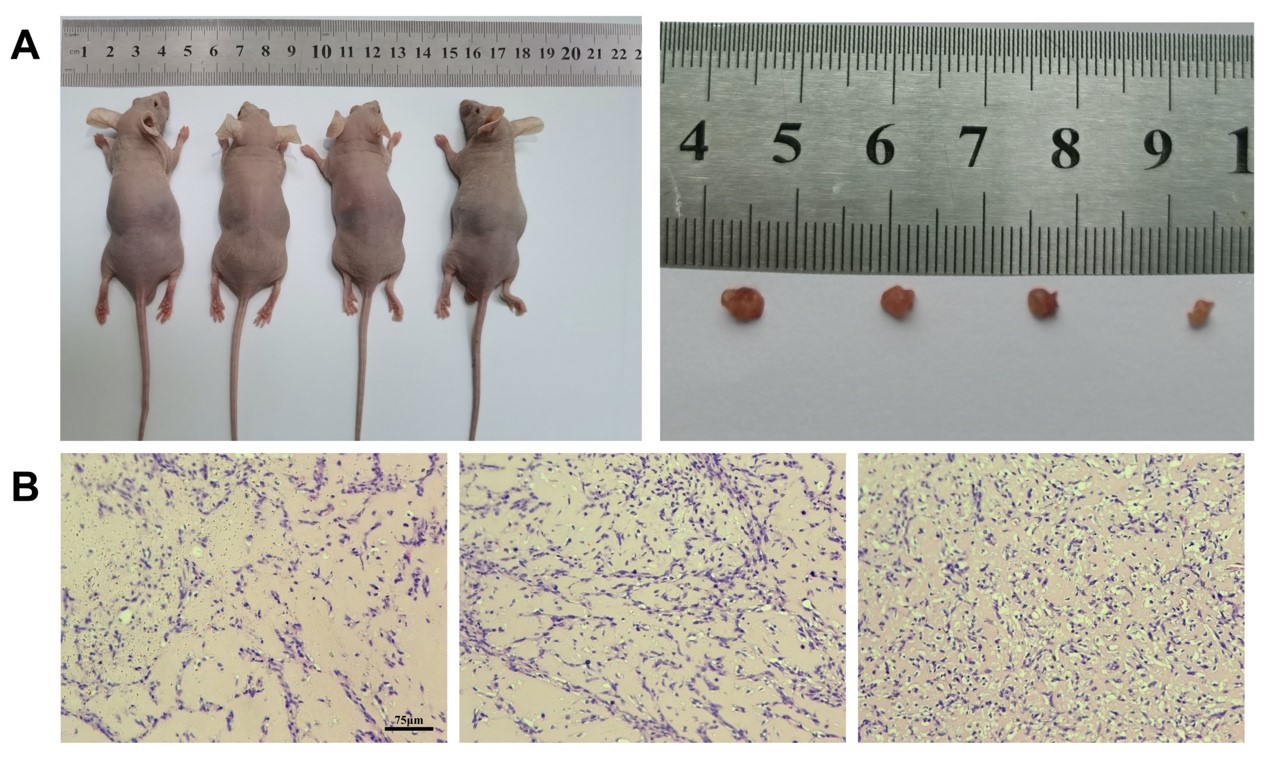

Supplement: Supplementary file 1 — Data S1. [file JCMM-28-e70200-s001.zip › jcmm70200-sup-0002-Supplementary Figure 2.jpg]

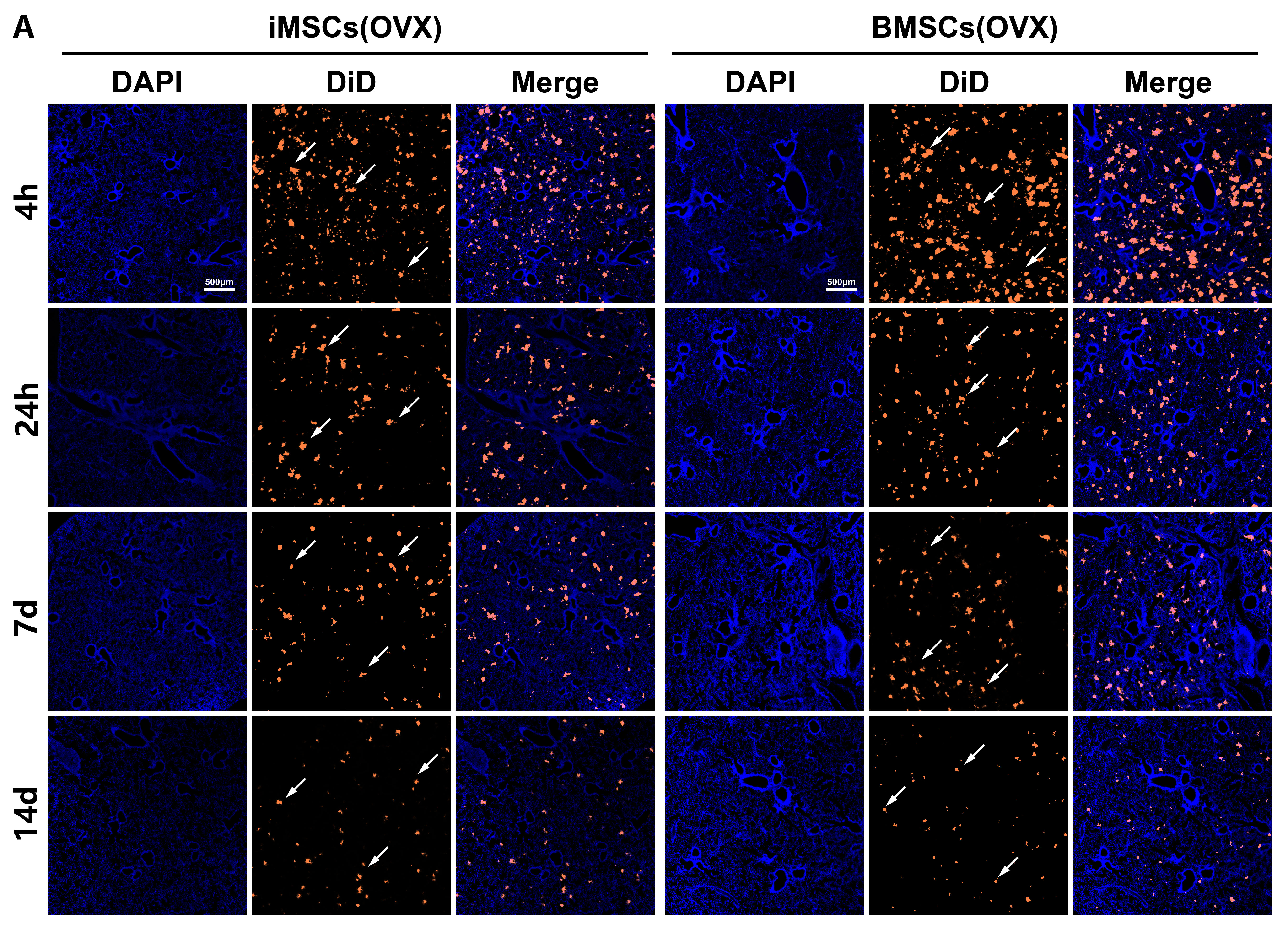

Supplement: Supplementary file 1 — Data S1. [file JCMM-28-e70200-s001.zip › jcmm70200-sup-0003-Supplementary Figure 3A.jpg]

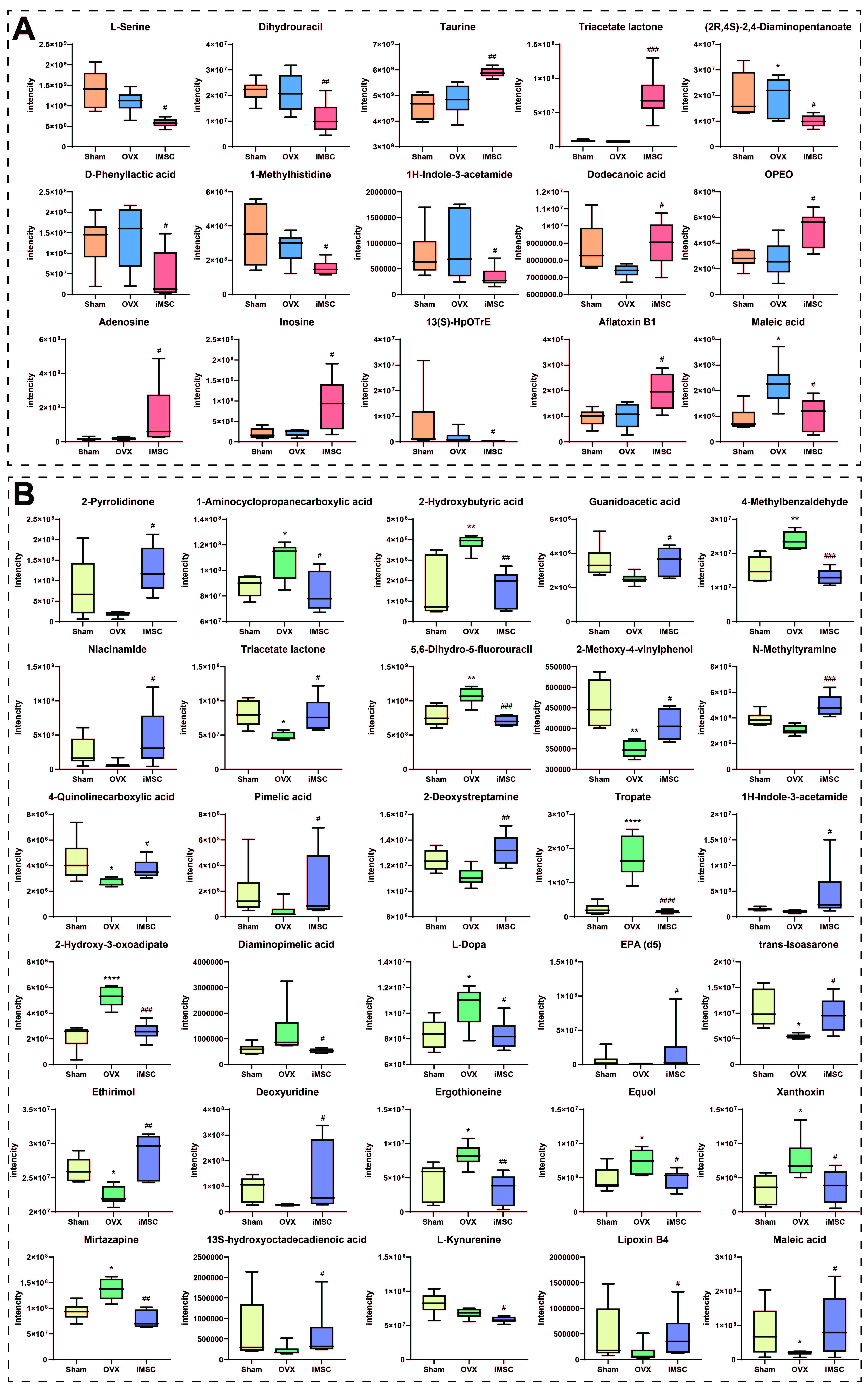

Supplement: Supplementary file 1 — Data S1. [file JCMM-28-e70200-s001.zip › jcmm70200-sup-0004-Supplementary Figure 4.jpg]

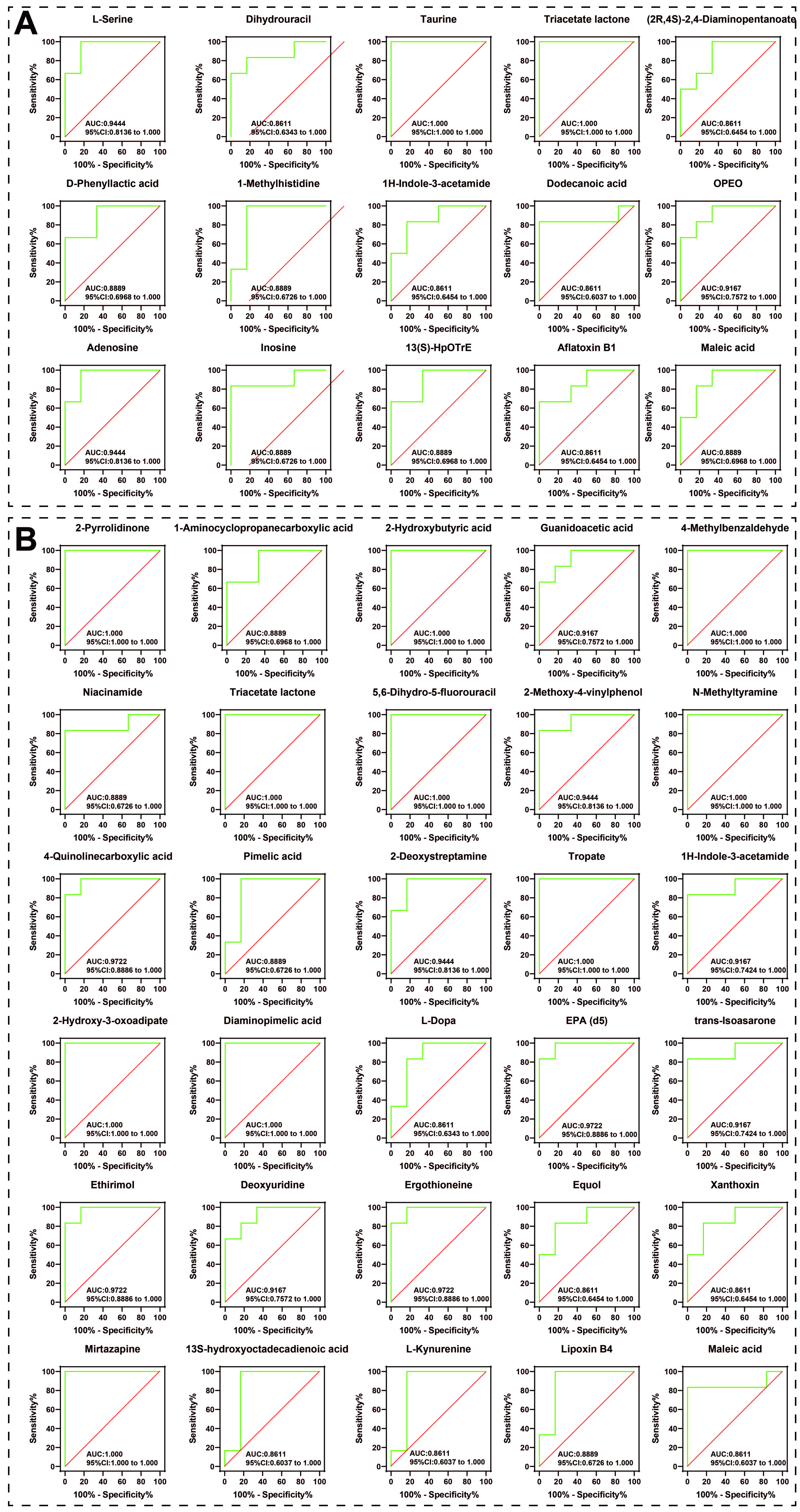

Supplement: Supplementary file 1 — Data S1. [file JCMM-28-e70200-s001.zip › jcmm70200-sup-0005-Supplementary Figure 5.jpg]

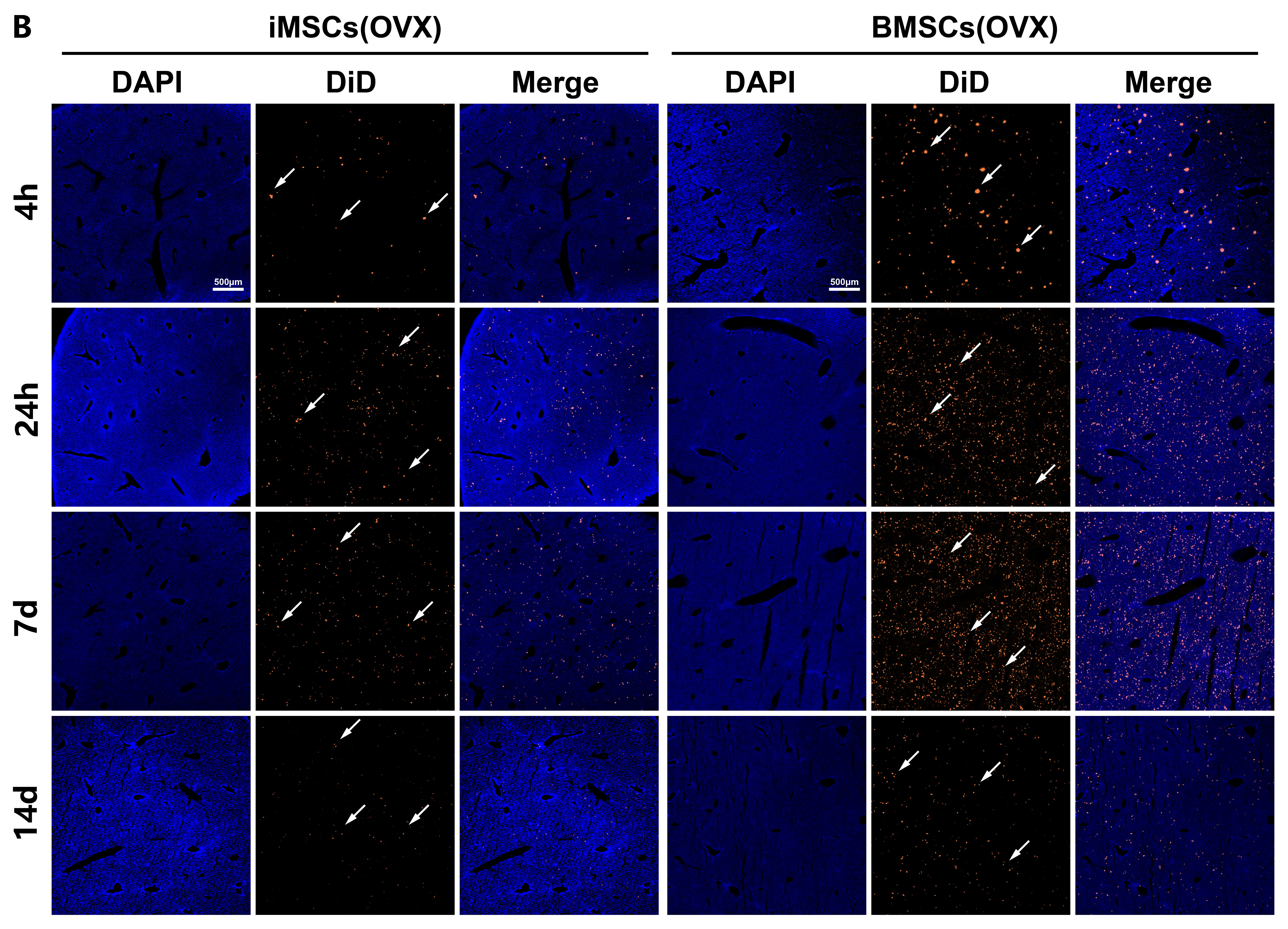

Supplement: Supplementary file 1 — Data S1. [file JCMM-28-e70200-s001.zip › Supplementary Figure 3B.jpg]

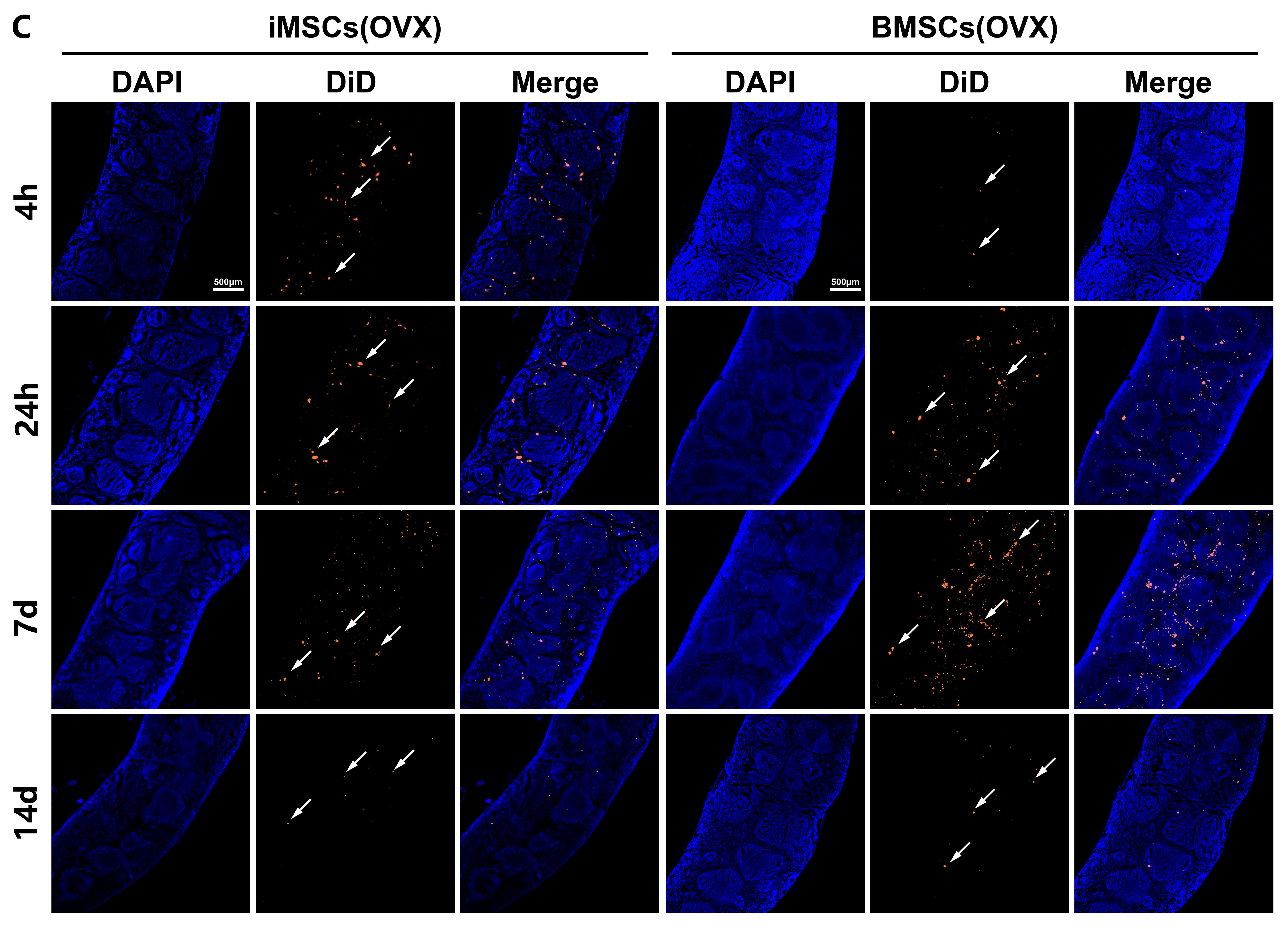

Supplement: Supplementary file 1 — Data S1. [file JCMM-28-e70200-s001.zip › Supplementary Figure 3C.jpg]

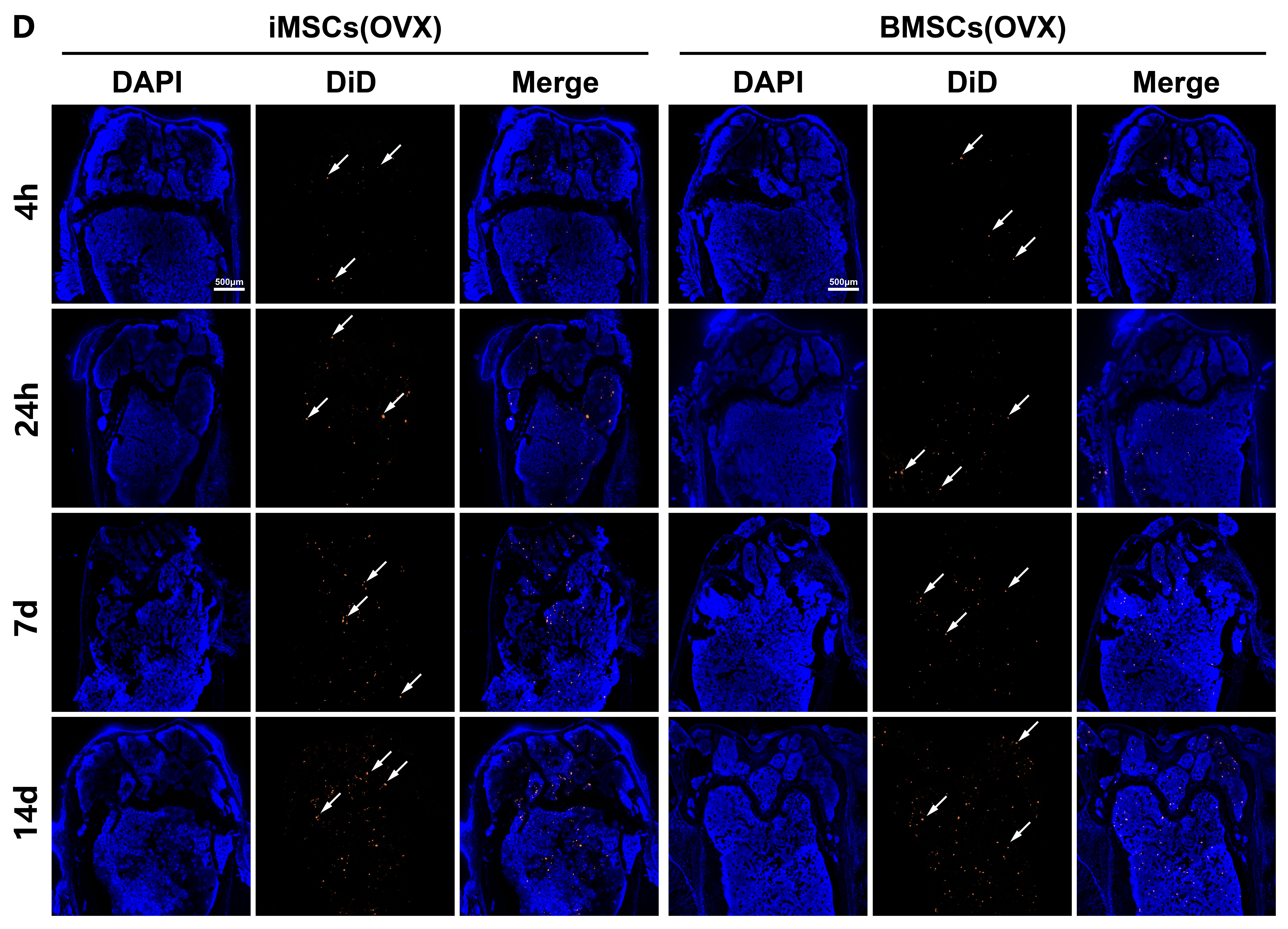

Supplement: Supplementary file 1 — Data S1. [file JCMM-28-e70200-s001.zip › Supplementary Figure 3D.jpg]

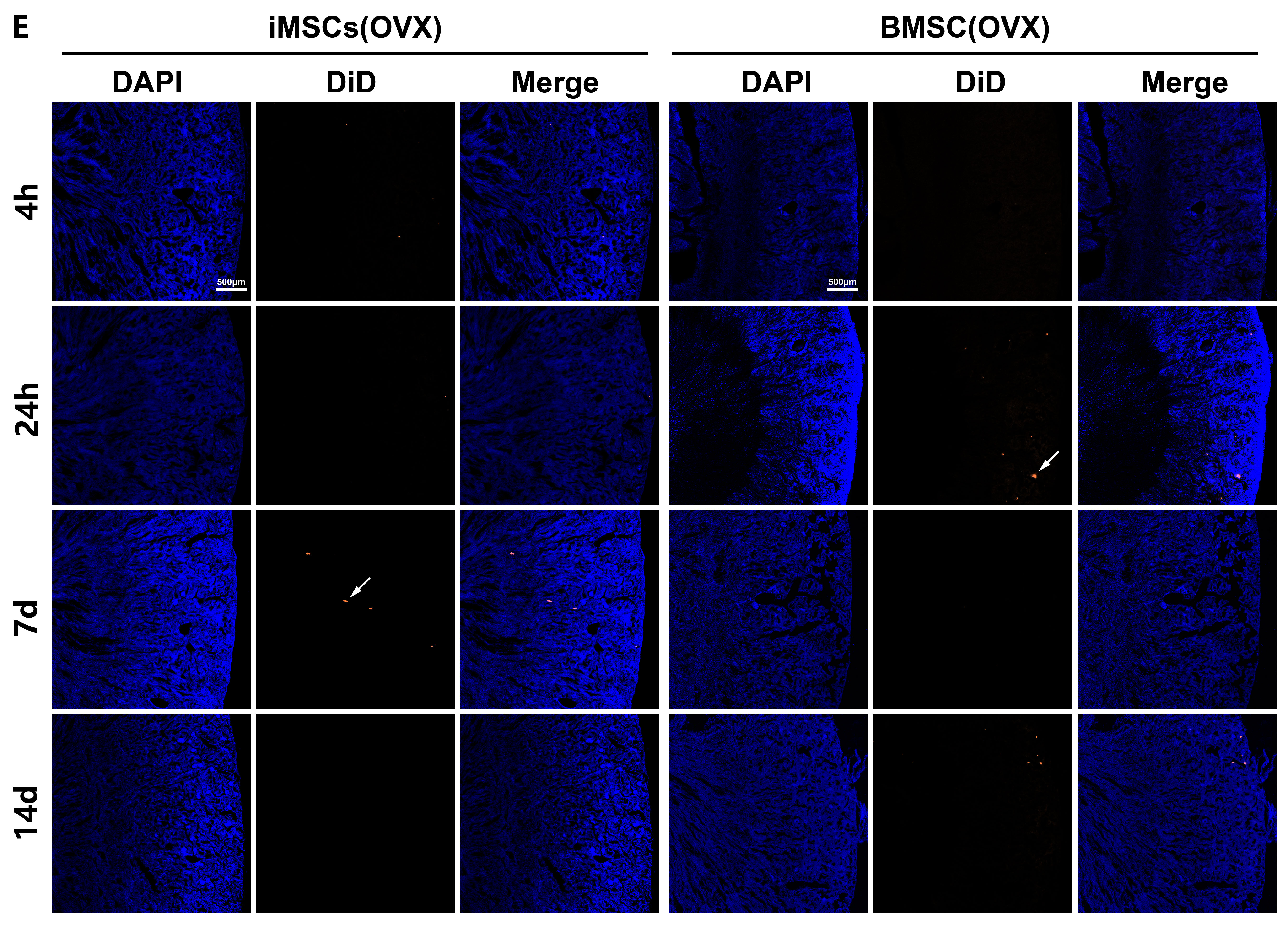

Supplement: Supplementary file 1 — Data S1. [file JCMM-28-e70200-s001.zip › Supplementary Figure 3E.jpg]

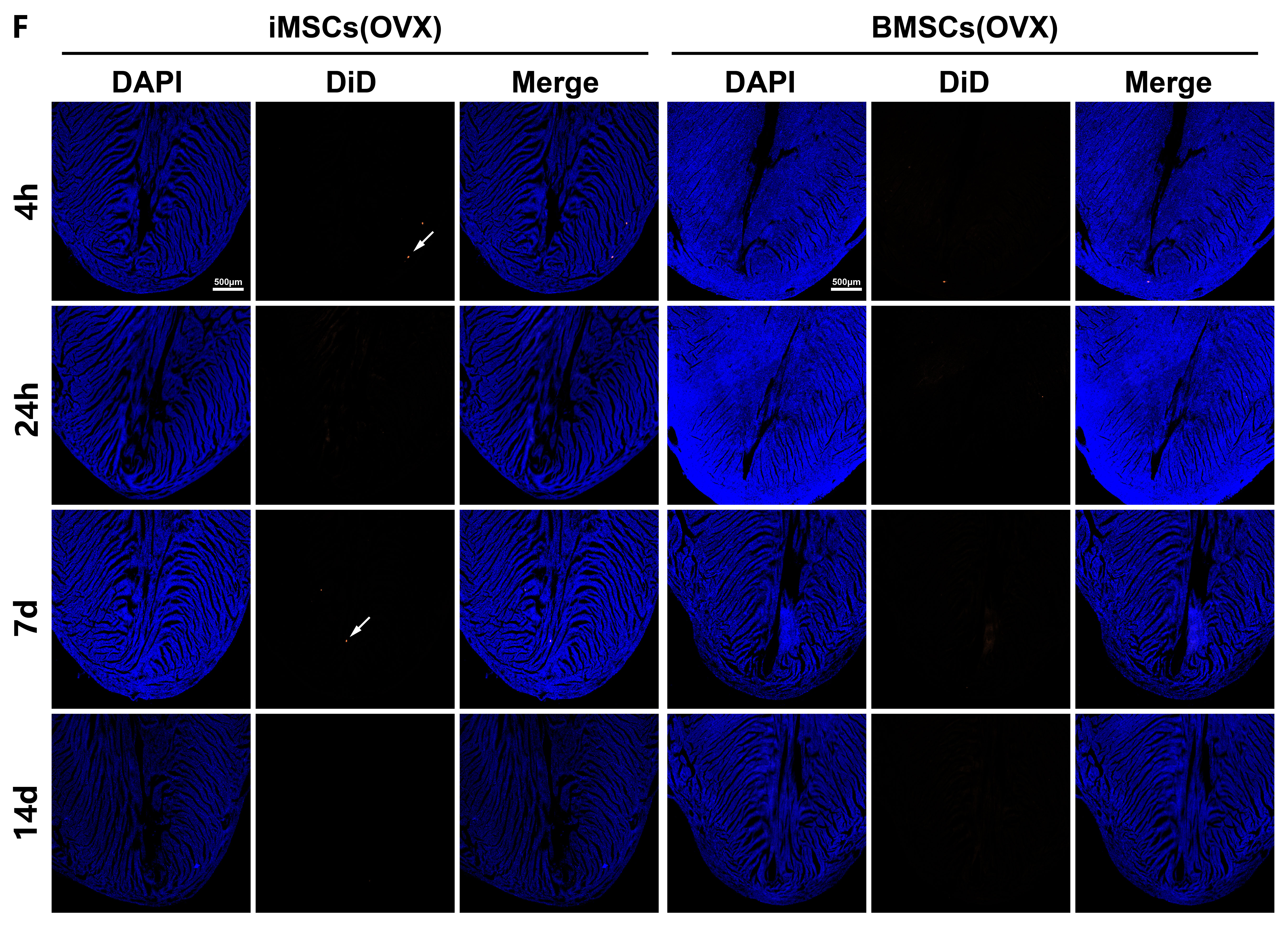

Supplement: Supplementary file 1 — Data S1. [file JCMM-28-e70200-s001.zip › Supplementary Figure 3F.jpg]

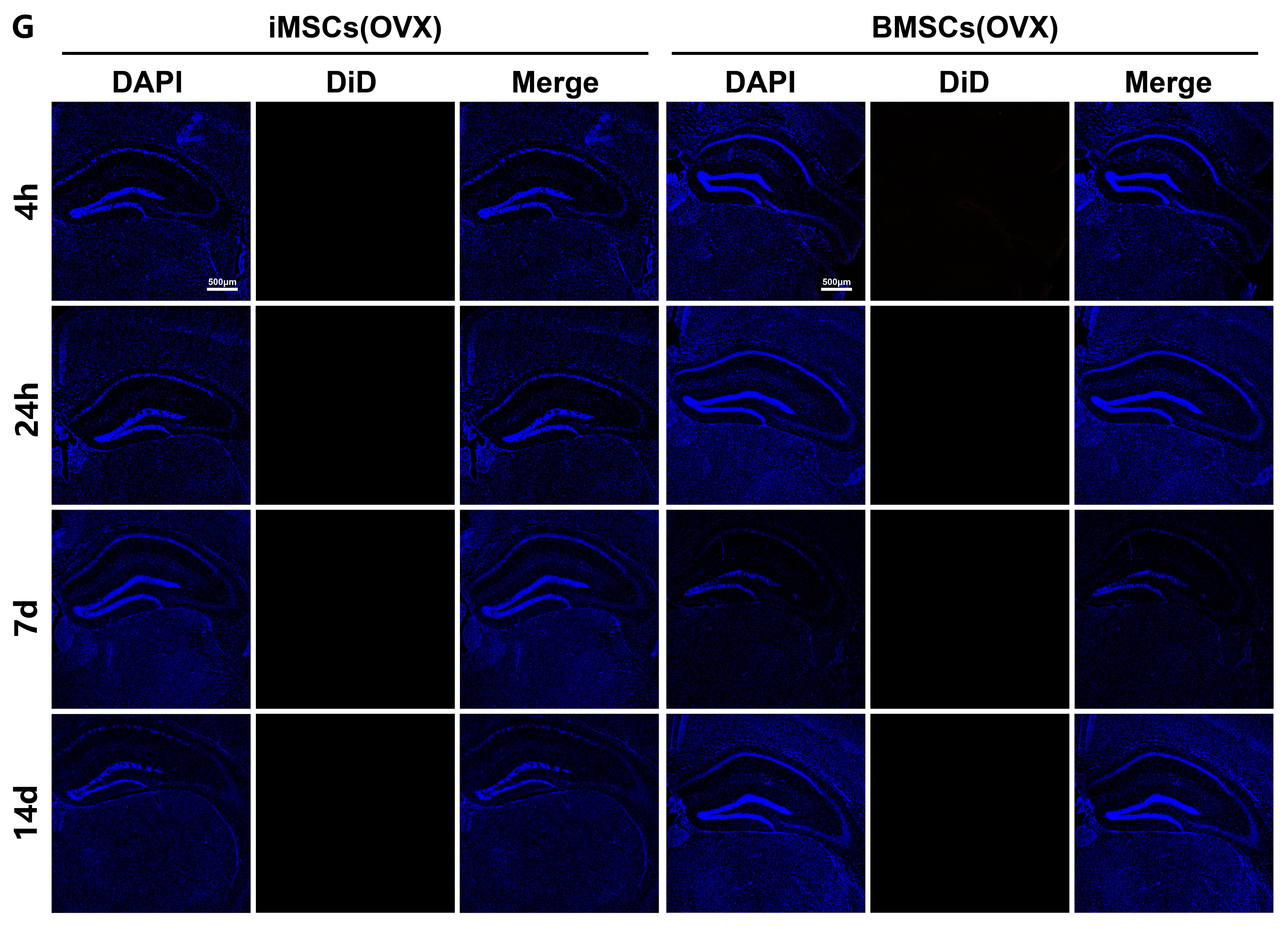

Supplement: Supplementary file 1 — Data S1. [file JCMM-28-e70200-s001.zip › Supplementary Figure 3G.jpg]

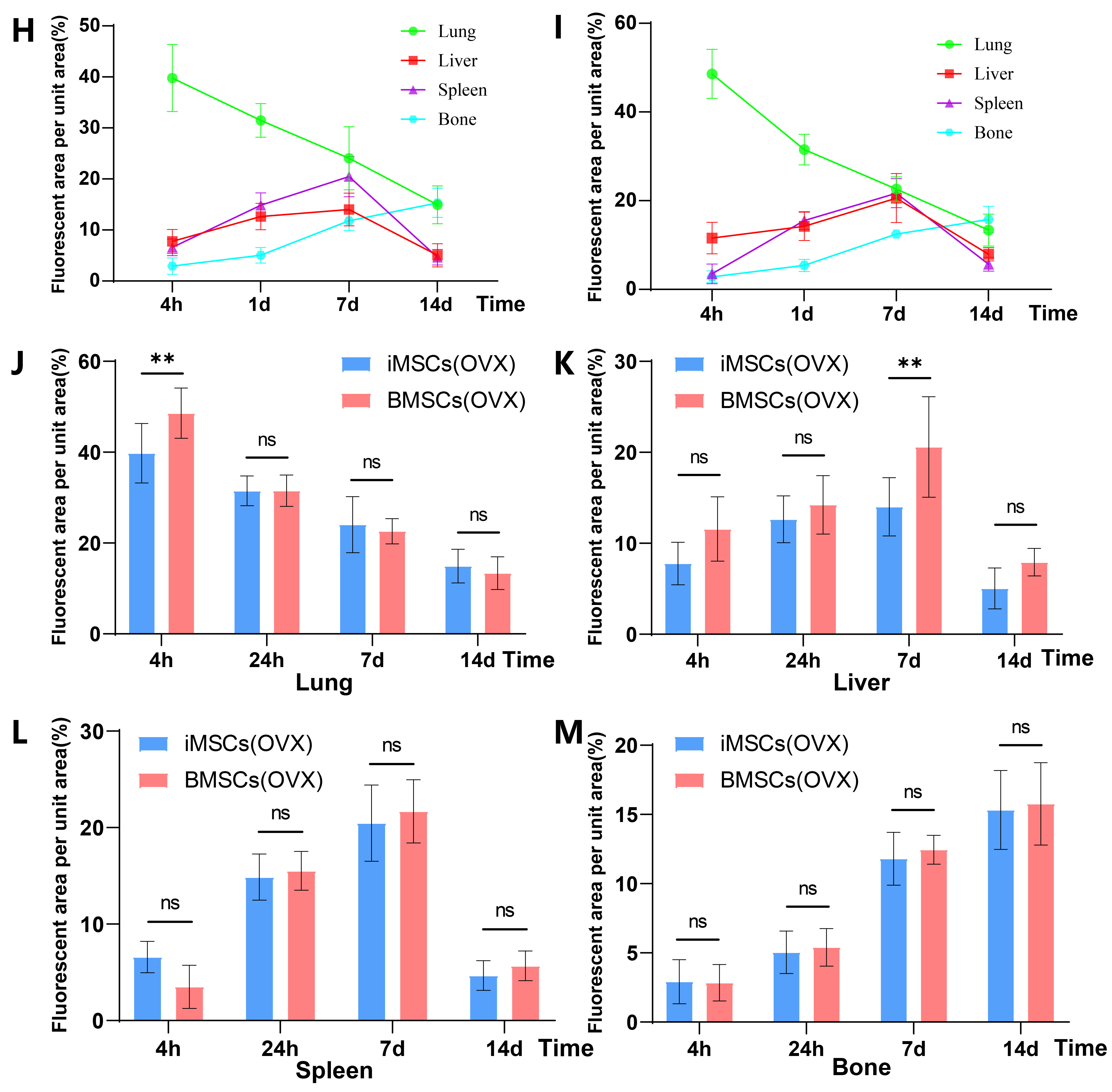

Supplement: Supplementary file 1 — Data S1. [file JCMM-28-e70200-s001.zip › Supplementary Figure 3H-M.jpg]
